# Physiological and Agronomic Responses of Maize (*Zea mays* L.) to Compost and PGPR Under Different Salinity Levels

**DOI:** 10.3390/plants14101539

**Published:** 2025-05-20

**Authors:** Ibrahim El-Akhdar, Nevien Elhawat, Mahmoud M. A. Shabana, Hesham M. Aboelsoud, Tarek Alshaal

**Affiliations:** 1 Department of Microbiology, Soils, Water and Environment Research Institute (SWERI), Agriculture Research Center (ARC), Giza 12112, Egypt; dr.elakhdar@yahoo.com; 2 Institute of Applied Plant Biology, Faculty of Agricultural and Food Sciences and Environmental Management, University of Debrecen, 4032 Debrecen, Hungary; alshaal.tarek@agr.unideb.hu; 3 Faculty of Agriculture (for Girls), Al-Azhar University, Nasr City, Cairo 11884, Egypt; 4 Soils, Water and Environment Research Institute (SWERI), Agricultural Research Center (ARC), Giza 12619, Egypt; shabanamma@gmail.com; 5 Soil Improvement and Conservation Research Department, Soils, Water, and Environment Research Institute (SWERI), Agriculture Research Center (ARC), Giza 12112, Egypt; hm.aboelsoud@gmail.com; 6 Soil and Water Science Department, Faculty of Agriculture, Kafrelsheikh University, Kafr El-Sheikh 33516, Egypt

**Keywords:** salinity stress, maize yield, compost, PGPR, soil health, nitrogen uptake, cultivar

## Abstract

Salinity stress severely limits maize (*Zea mays* L.) productivity, necessitating sustainable mitigation strategies to ensure food security in affected regions. This study investigates the efficacy of compost (5 and 10 t/ha) and plant growth-promoting rhizobacteria (PGPR; *Azospirillum brasilense*) in enhancing maize productivity and soil health under salinity stress (EC_e_ 3.5 and 6.3 dS/m) across three varieties (Single Cross 131, 132, and 178) in field experiments conducted in 2023 and 2024. Combined compost-10 + PGPR treatment significantly increased grain yield by up to 197% and straw yield by nearly 300% in Single Cross 178 under high salinity, surpassing single treatments. Nitrogen content in grains and straw rose by 157%, while proline, peroxidase activity, and chlorophyll content improved, indicating robust stress tolerance. Soil properties, including pH, EC_e_, sodium adsorption ratio, and exchangeable sodium percentage, were significantly ameliorated, with bulk density reduced and porosity increased. Soil organic matter and microbial populations (bacteria and fungi) were also enhanced. Single Cross 178 exhibited superior stress tolerance, highlighting varietal differences. These findings, supported by comparisons with the existing literature, underscore the synergistic role of compost and PGPR in improving nutrient uptake, antioxidant defenses, and soil structure. This study offers a sustainable strategy for maize cultivation in saline environments, with implications for global food security.

## 1. Introduction

Soil salinity is a major abiotic stress that significantly limits agricultural productivity, particularly in arid and semi-arid regions [1]. High salinity levels disrupt plant physiological processes, reduce nutrient uptake, and impair growth, ultimately leading to decreased crop yields [2]. Maize (*Zea mays* L.), a globally important cereal crop, is particularly sensitive to salinity stress, which affects its grain yield, straw yield, and overall physiological health [3]. The sensitivity of maize to salinity is attributed to its relatively low tolerance to osmotic stress and ion toxicity, which can lead to reduced photosynthetic efficiency, impaired water relations, and altered nutrient balance [4]. As global food demand continues to rise, particularly in regions prone to salinity, there is an urgent need to develop sustainable strategies to improve crop productivity under such adverse conditions.

To mitigate the adverse effects of salinity, sustainable agricultural practices, such as the application of organic amendments like compost, have gained considerable attention [5]. Compost application has been shown to improve soil structure, enhance nutrient availability, and increase microbial activity, all of which contribute to better plant growth under stress conditions [6]. Compost not only provides essential macro- and micronutrients but also improves soil water retention and reduces the bioavailability of toxic ions such as sodium (Na^+^) and chloride (Cl⁻), which are detrimental to plant growth under saline conditions [7]. Furthermore, compost enhances the soil’s cation-exchange capacity (CEC), which helps in mitigating the negative effects of salinity by promoting the uptake of beneficial nutrients like potassium (K^+^) and calcium (Ca^2+^) [8]. The organic matter in compost also acts as a buffer, stabilizing soil pH and reducing the impact of salinity on soil microbial communities [7].

In addition to compost, plant growth-promoting rhizobacteria (PGPR), a group of beneficial soil bacteria, play a crucial role in promoting plant growth by enhancing nutrient uptake, producing phytohormones, and inducing systemic resistance against abiotic stresses [8,9,10,11,12]. PGPR can alleviate salinity stress by improving root architecture, increasing the production of osmoprotectants such as proline, and enhancing antioxidant enzyme activity, which collectively help plants cope with oxidative stress induced by high salinity [13]. For instance, PGPR strains such as *Azospirillum* and *Azotobacter* have been reported to produce exopolysaccharides that bind Na^+^ ions, reducing their uptake by plants and thereby mitigating ion toxicity [14]. Moreover, PGPR can modulate the expression of stress-responsive genes, enhancing the plant’s ability to maintain osmotic balance and cellular homeostasis under saline conditions [15]. The ability of PGPR to solubilize phosphate, fix nitrogen, and produce siderophores further contributes to improved nutrient availability and uptake, which are critical for plant growth under stress [16].

The combined use of compost and PGPR has been reported to synergistically improve crop performance under saline conditions by enhancing soil health and plant resilience [17]. Compost provides a favorable environment for PGPR colonization and activity, while PGPR, in turn, enhances the mineralization of organic matter in compost, making nutrients more available to plants [18]. This mutualistic interaction can lead to improved soil fertility, enhanced microbial diversity, and better plant growth under salinity stress. For example, studies have shown that the combined application of compost and PGPR significantly increases the biomass and yield of crops such as wheat and maize under saline conditions [8]. The synergistic effects of compost and PGPR are particularly evident in their ability to improve soil aggregation, water infiltration, and root penetration, all of which are critical for plant growth in saline soils [6].

Salinity stress triggers various physiological and biochemical responses in plants, including the accumulation of osmolytes like proline, the increased activity of antioxidant enzymes such as peroxidase, and alterations in chlorophyll content [19,20]. These responses are critical for maintaining cellular homeostasis and protecting plants from oxidative damage under stress [21]. Proline, for instance, acts as an osmoprotectant, stabilizing proteins and cellular structures, while antioxidant enzymes such as peroxidase scavenge reactive oxygen species (ROS) that accumulate under salinity stress [22]. Chlorophyll content, a key indicator of photosynthetic efficiency, often declines under salinity due to the degradation of chlorophyll pigments and the inhibition of chlorophyll synthesis [12]. Understanding these physiological responses is essential for developing strategies to enhance plant tolerance to salinity.

Furthermore, salinity affects soil microbial communities, which play a vital role in nutrient cycling and plant health. High salinity levels can reduce microbial diversity and activity, leading to impaired nutrient cycling and reduced plant growth [23]. However, the application of compost and PGPR can mitigate these negative effects by promoting the growth of beneficial microbial populations and enhancing their functional roles in the soil ecosystem [18]. For example, compost provides a carbon-rich substrate that supports the growth of salt-tolerant microbial communities, while PGPR can enhance the activity of nitrogen-fixing and phosphate-solubilizing bacteria, improving nutrient availability for plants [16]. The interaction between compost, PGPR, and soil microbial communities is a key factor in determining the success of sustainable agricultural practices in saline environments.

Despite the growing body of research on the individual effects of compost and PGPR, there is limited information on their combined impact on maize varieties under different salinity levels. This study aims to investigate the synergistic effects of compost and PGPR on maize productivity and resilience under saline soil conditions, with the goal of developing sustainable agricultural strategies to mitigate salinity stress. Specifically, the objectives are: (1) to evaluate the efficacy of compost (applied at 5 and 10 t/ha), PGPR (*Azospirillum brasilense*), and their combined applications in enhancing grain and straw yields, nitrogen uptake, and physiological stress responses (e.g., proline accumulation, peroxidase activity, and chlorophyll content) in three maize varieties (Single Cross 131, 132, and 178) grown under two salinity levels (EC_e_ 3.5 and 6.3 dS/m); (2) to assess the mechanisms by which compost and PGPR improve soil health, including reductions in soil electrical conductivity (EC_e_), sodium adsorption ratio (SAR), and exchangeable sodium percentage (ESP), as well as increases in soil organic matter (SOM), available nitrogen (Ava-N), and microbial populations, thereby alleviating salinity-induced constraints; (3) to elucidate the synergistic interactions between compost and PGPR in promoting nutrient availability, soil structure, and microbial activity, and to determine how these interactions enhance maize tolerance to salinity stress through improved osmotic adjustment, antioxidant defenses, and photosynthetic efficiency; and (4) to identify varietal differences in salinity tolerance among maize genotypes and their responsiveness to compost and PGPR treatments, highlighting the role of genetic factors in optimizing amendment efficacy for sustainable crop production in saline environments.

## 2. Results

### 2.1. Maize Yield and Nitrogen Response to Compost and PGPR Treatments Under Salinity Stress

The grain yield was significantly improved by the applied treatments under both salinity levels (Table 1). At EC_e_ 3.5 dS/m, the highest grain yields were achieved with compost-10 + PGPR, ranging from 10.7 to 11.6 t/ha across the three maize varieties, representing a substantial increase compared to the control. The Single Cross 178 cultivar showed the highest grain yield in 2023 and 2024 under the EC_e_ 3.5, recording 12.0 and 12.1 t/ha, respectively. Under the more severe EC_e_ 6.3 dS/m condition, the same treatment maintained superior performance, with yields reaching 8.9–10.5 t/ha. Among the varieties, Single Cross 178 showed the most pronounced response, particularly under high salinity, where compost-10 + PGPR enhanced grain yield by up to 197% compared to the untreated control.

Straw yield followed a similar trend, with the combined treatments (compost-5 + PGPR and compost-10 + PGPR) producing the highest values. At EC_e_ 3.5 dS/m, straw yields peaked at 21.7–25.5 t/ha with compost-10 + PGPR, while at EC_e_ 6.3 dS/m, they reached 18.0–19.8 t/ha. Single Cross 178 again demonstrated the greatest improvement, particularly under high salinity, where straw yield nearly tripled with the best-performing treatments compared to the control.

Nitrogen content in grains (N-grain) increased consistently with the application of compost and PGPR. The highest values were recorded with compost-10 + PGPR, ranging from 1.34 to 1.36% at EC_e_ 3.5 dS/m and 1.15 to 1.26% at EC_e_ 6.3 dS/m. Single Cross 131 and 132 showed moderate but significant improvements, while Single Cross 178 exhibited the most notable enhancement, particularly under high salinity stress.

Nitrogen content in straw (N-straw) also improved with the treatments, particularly under combined applications. The highest N-straw values were observed with compost-10 + PGPR, reaching 2.23–2.24% at EC_e_ 3.5 dS/m and 1.99–2.09% at EC_e_ 6.3 dS/m. Single Cross 178 displayed the strongest response, with N-straw levels increasing by up to 157% compared to the control under high salinity. These results highlight the effectiveness of compost and PGPR in enhancing nitrogen uptake, particularly under stress conditions.

Varietal differences were evident across all of the measured traits. Single Cross 178 consistently outperformed the other varieties, demonstrating greater tolerance to salinity and a stronger response to the treatments. Single Cross 131 and 132 also benefited from the amendments but to a lesser extent, suggesting that genetic factors play a key role in stress adaptation. The superior performance of the combined treatments underscores their potential as a sustainable strategy for improving maize productivity in saline soils.

### 2.2. Proline Content, Peroxidase Activity, and Total Chlorophyll in Three Maize Varieties Under Different Salinity Levels and Treatments

The proline content in maize leaves showed a clear response to salinity stress and applied treatments (Table 2). Under EC_e_ 3.5 dS/m, proline levels remained relatively low across all varieties, ranging from 2.25 to 4.23 μmol/g FW, with the highest values observed in the compost-5 + PGPR treatments. However, under severe salinity (EC_e_ 6.3 dS/m), proline accumulation increased dramatically, reaching 19.05–37.37 μmol/g FW, indicating its role as an osmoprotectant. The Single Cross 131 variety consistently showed the highest proline accumulation under stress, particularly with the compost-10 + PGPR treatment (37.37 μmol/g FW in 2023), suggesting stronger osmotic adjustment capability compared to the varieties Single Cross 132 and Single Cross 178.

Peroxidase activity followed a similar pattern, with significantly higher values under EC_e_ 6.3 dS/m compared to EC_e_ 3.5 dS/m across all varieties. The enzyme activity ranged from 12.32 to 39.42 μmol H_2_O_2_/min/g FW at EC_e_ 3.5 dS/m, while at EC_e_ 6.3 dS/m it increased to 45.03–180.95 μmol H_2_O_2_/min/g FW. The Single Cross 131 variety demonstrated the highest peroxidase activity under both salinity levels, particularly with PGPR and compost + PGPR combinations, reaching up to 180.95 μmol H_2_O_2_/min/g FW in 2023 with the compost-10 + PGPR treatment. This suggests more efficient antioxidant defense mechanisms in the Single Cross 131 variety compared to the other two varieties.

The total chlorophyll content was negatively affected by salinity stress, with lower values observed at EC_e_ 6.3 dS/m (0.51–1.55 mg/g FW) compared to EC_e_ 3.5 dS/m (1.36–2.94 mg/g FW). Among the treatments, compost applications, especially when combined with PGPR, helped maintain higher chlorophyll levels under stress conditions. The Single Cross 131 variety maintained the highest chlorophyll content under both salinity levels, with the compost-10 + PGPR treatment showing the best results (2.94 mg/g FW at EC_e_ 3.5 dS/m and 1.55 mg/g FW at EC_e_ 6.3 dS/m in 2024). The chlorophyll preservation pattern followed Single Cross 131 > Single Cross 132 > Single Cross 178, indicating differential sensitivity to salinity among the varieties.

The results demonstrate that combined applications of compost and PGPR were generally more effective than single treatments in mitigating salinity stress effects on maize. The Single Cross 131 variety consistently showed better stress-tolerance indicators compared to the Single Cross 132 and Single Cross 178 varieties, as evidenced by higher proline accumulation, peroxidase activity, and chlorophyll preservation under salinity stress. These findings suggest that genotype-specific responses should be considered when developing salinity mitigation strategies for maize cultivation.

### 2.3. Impact of Compost and PGPR Treatments on Soil Properties of Maize Cultivation Under Salinity Stress

The soil pH measurements showed consistent reductions across all varieties when treated with compost and PGPR applications (Table 3). Under EC_e_ 3.5 dS/m, pH values ranged from 7.30 to 7.92 in control plots, decreasing to 7.30–7.80 with the compost treatments. More pronounced effects were observed under EC_e_ 6.3 dS/m, where compost-10 + PGPR treatment reduced pH from 8.22–8.45 in controls to 7.70–7.90 across varieties. The Single Cross 178 variety exhibited the greatest pH reduction, particularly with the compost-10 + PGPR treatment (7.82 in 2023 at EC_e_ 6.3 dS/m), suggesting better soil amendment efficiency compared to the Single Cross 132 and Single Cross 178 varieties.

The EC_e_ showed significant decreases with all of the treatments compared to the controls. At EC_e_ 3.5 dS/m, the EC_e_ values dropped from 4.10–5.82 dS/m in the controls to 2.14–3.72 dS/m with the compost-10 + PGPR treatment. Under EC_e_ 6.3 dS/m, the most effective treatment (compost-10 + PGPR) reduced EC_e_ from 6.22–7.67 dS/m in controls to 2.30–4.21 dS/m. The Single Cross 132 variety demonstrated the most consistent EC_e_ reductions across both years, with the compost-10 + PGPR treatment achieving the lowest values (2.14 dS/m at EC_e_ 3.5 dS/m and 3.87 dS/m at EC_e_ 6.3 dS/m in 2023).

The SAR followed similar improvement patterns, with the compost-10 + PGPR treatment showing the greatest reductions. At EC_e_ 3.5 dS/m, the SAR decreased from 10.14–12.09 in controls to 8.07–9.10 with compost-10 + PGPR. Under EC_e_ 6.3 dS/m, the SAR values dropped from 13.00–14.44 in controls to 9.16–10.50 with the same treatment. The Single Cross 131 variety showed the highest baseline SAR values but also the most significant improvements, particularly under EC_e_ 6.3 dS/m where compost-10 + PGPR reduced the SAR by 26.5% compared to the control.

The ESP mirrored the SAR trends, with compost-10 + PGPR consistently producing the lowest values across both salinity levels (Table 4). At EC_e_ 3.5 dS/m, ESP decreased from 12.04–14.21% in controls to 9.62–10.84% with compost-10 + PGPR. Under EC_e_ 6.3 dS/m, the treatment reduced ESP from 15.19–16.70% in controls to 10.91–12.46%. Variety 3 exhibited the most notable ESP reductions, particularly under EC_e_ 6.3 dS/m where compost-5 + PGPR treatment lowered ESP by 33.7% compared to the control.

The results demonstrate that combined compost and PGPR applications were more effective than single treatments in improving soil properties under salinity stress. Compost-10 + PGPR consistently showed the best performance across all of the measured parameters. While all of the varieties responded positively to the treatments, the Single Cross 178 variety generally showed the greatest improvements in soil conditions, followed by the Single Cross 132 variety and then the Single Cross 131 variety. These findings highlight the potential of integrated soil amendments in mitigating salinity effects in maize cultivation, with varietal differences playing a significant role in treatment efficacy.

The soil bulk density measurements showed consistent improvements with compost and PGPR applications across all varieties. Under EC_e_ 3.5 dS/m, bulk density decreased from the control values of 1.36–1.37 g/cm^3^ to 1.28–1.34 g/cm^3^ with the compost treatments, with the lowest values observed in compost-10 + PGPR applications. The effects were more pronounced under EC_e_ 6.3 dS/m, where bulk density reductions ranged from 1.41–1.45 g/cm^3^ in the controls to 1.33–1.36 g/cm^3^ with the treatments. The Single Cross 132 variety exhibited the most significant bulk density improvements, particularly with the compost-10 + PGPR treatment achieving 1.27 g/cm^3^ at EC_e_ 3.5 dS/m and 1.33 g/cm^3^ at EC_e_ 6.3 dS/m in 2024, indicating better soil structure development compared to the Single Cross 131 and Single Cross 178 varieties.

Total porosity showed corresponding increases with the applied treatments, demonstrating an inverse relationship with bulk density changes. At EC_e_ 3.5 dS/m, porosity increased from the control values of 45.52–48.17% to 49.45–52.07% with the compost-10 + PGPR treatment. Under EC_e_ 6.3 dS/m conditions, similar improvements were observed, with porosity rising from 45.15–46.66% in the controls to 48.69–49.81% with the treatments. The Single Cross 132 variety, again, showed the most notable enhancements, reaching 52.07% porosity at EC_e_ 3.5 dS/m with compost-10 + PGPR in 2024, suggesting superior soil aeration and root-zone conditions compared to the other varieties.

The results demonstrate that combined compost and PGPR applications were more effective than single treatments in improving soil physical properties under salinity stress. Compost-10 + PGPR consistently provided the best results across both of the measured parameters. While all of the varieties responded positively to the treatments, the Single Cross 132 variety showed the greatest improvements in soil physical conditions, followed by the Single Cross 178 variety and then the Single Cross 131 variety. These improvements in bulk density and porosity are particularly important for maize growth under salinity stress, as they enhance root development, water infiltration, and gas exchange in the rhizosphere. The findings highlight the potential of integrated organic amendments and microbial inoculants to mitigate salinity-induced soil compaction and improve overall soil health for maize production.

### 2.4. Changes in Soil Organic Matter (SOM), Available Nitrogen (Ava-N), Total Bacteria, and Total Fungi Under Different Treatments and Salinity Levels

The SOM content showed consistent improvements with compost applications across all varieties and salinity levels (Table 5). Under EC_e_ 3.5 dS/m, SOM increased from the control values of 1.04–1.08% to 1.21–1.28% with the compost treatments, with the highest values observed in compost-10 + PGPR applications. Similar improvements were seen under EC_e_ 6.3 dS/m, where SOM rose from 0.84–0.86% in the controls to 1.15–1.21% with the treatments. The Single Cross 132 variety exhibited the most significant SOM increases, particularly with the compost-10 + PGPR treatment, reaching 1.27% at EC_e_ 3.5 dS/m and 1.21% at EC_e_ 6.3 dS/m in 2024, indicating better organic matter retention compared to the other varieties.

The available nitrogen (Ava-N) levels demonstrated substantial enhancements with all of the treatments, particularly with the combined compost and PGPR applications. At EC_e_ 3.5 dS/m, Ava-N increased from 19.3–20.7 ppm in the controls to 35.6–40.6 ppm with the compost-10 + PGPR treatment. Under EC_e_ 6.3 dS/m conditions, similar improvements were observed, with Ava-N rising from 15.6–17.0 ppm in the controls to 32.7–37.3 ppm with the treatments. The Single Cross 131 variety showed the highest Ava-N levels under both salinity conditions, reaching 40.5 ppm at EC_e_ 3.5 dS/m with compost-10 + PGPR in 2024, suggesting superior nitrogen mineralization and availability compared to the Single Cross 132 and Single Cross 178 varieties.

The total bacterial populations were significantly enhanced by the PGPR-containing treatments across all varieties. At EC_e_ 3.5 dS/m, bacterial counts increased from 3.3–4.5 × 10^7^ CFU/g in the controls to 5.4–7.3 × 10^7^ CFU/g with the compost-5 + PGPR treatment. Under EC_e_ 6.3 dS/m, similar increases were observed, with counts rising from 2.9–3.9 × 10^7^ CFU/g in the controls to 4.8–5.5 × 10^7^ CFU/g with the treatments. The Single Cross 131 variety consistently maintained the highest bacterial populations, particularly with the compost-5 + PGPR treatment achieving 7.3 × 10^7^ CFU/g at EC_e_ 3.5 dS/m in 2024, indicating a more favorable environment for microbial growth compared to the other varieties.

The total fungal populations followed similar enhancement patterns, with the greatest increases observed in the combined compost and PGPR treatments. At EC_e_ 3.5 dS/m, the fungal counts rose from 1.3–1.7 × 10^5^ CFU/g in the controls to 1.7–2.0 × 10^5^ CFU/g with the treatments. Under EC_e_ 6.3 dS/m, the counts increased from 1.1–1.4 × 10^5^ CFU/g in the controls to 1.4–1.7 × 10^5^ CFU/g with the treatments. The Single Cross 178 variety showed the highest fungal populations under both salinity levels, reaching 2.0 × 10^5^ CFU/g at EC_e_ 3.5 dS/m with compost-10 + PGPR in 2024, suggesting better fungal community development compared to the Single Cross 131 and Single Cross 132 varieties.

The results demonstrate that the combined compost and PGPR applications were more effective than the single treatments in improving soil biological properties under salinity stress. Compost-10 + PGPR generally provided the best results for SOM and Ava-N, while compost-5 + PGPR showed superior performance for microbial populations. While all varieties responded positively to the treatments, the Single Cross 131 variety showed the greatest improvements in nitrogen availability and bacterial populations, the Single Cross 132 variety in organic matter content, and the Single Cross 178 variety in fungal development. These findings highlight the importance of integrated soil management strategies to enhance soil biological activity and nutrient cycling for maize production under saline conditions.

### 2.5. Pearson Correlation

**Figure 1:**
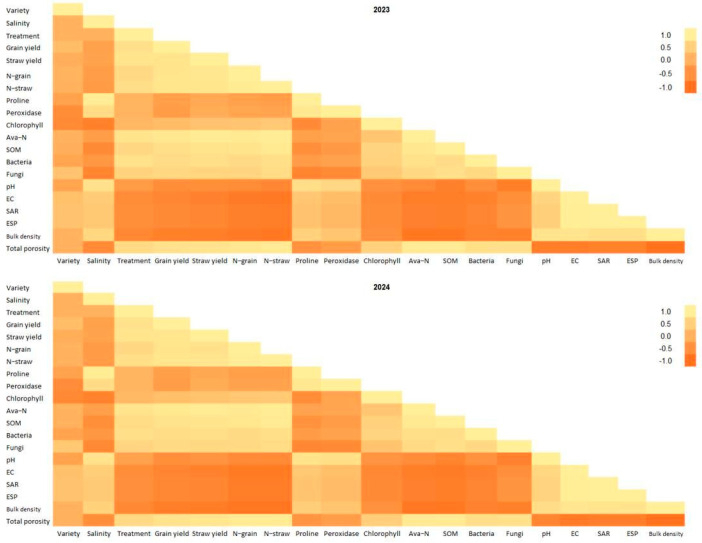
Pearson correlation matrices for maize growth and soil traits in 2023 and 2024, illustrating relationships among variety, salinity, treatments, and key parameters (grain yield, straw yield, N-grain, N-straw, proline, peroxidase, chlorophyll, available nitrogen, soil organic matter, microbial populations, pH, EC, SAR, ESP, bulk density, and total porosity) under salinity stress (EC_e_ 3.5 dS/m and 6.3 dS/m).

The results of the Pearson correlation highlighted the significant role of treatments, particularly compost-10 + PGPR, in enhancing maize growth and productivity by mitigating salinity stress, with notable variations among the varieties (Figure 1).

The grain yield showed strong positive correlations with the treatments (0.76 in 2023, 0.763 in 2024) and a negative correlation with salinity (−0.26 in 2023, −0.260 in 2024). Compost-10 + PGPR consistently increased yields, especially for Single cross 178, which outperformed others due to its stress tolerance. Single Cross 131 showed moderate yield improvements with compost-5 + PGPR, while Single Cross 132 had the lowest yields at EC_e_ 6.3 dS/m, indicating high salinity sensitivity. The treatments improved nutrient availability and soil structure, reducing yield losses.

Straw yield followed a similar pattern, with the treatments strongly correlated (0.82 in 2023, 0.818 in 2024) and salinity negatively impacting results (−0.22 in 2023, −0.222 in 2024). Compost-10 + PGPR significantly boosted straw yield, particularly for Single Cross 178, which maintained high biomass. Single Cross 131 benefited moderately from compost-5 + PGPR, whereas Single Cross 132 showed a reduced biomass with higher salinity, reflecting lower adaptability. The treatments enhanced water retention and microbial activity.

N-grain and N-straw contents were positively influenced by the treatments (0.64 and 0.73 in 2023, 0.618 and 0.737 in 2024, respectively) and negatively affected by salinity (−0.30 and −0.36 in 2023, −0.352 and −0.345 in 2024). Single Cross 178 exhibited the highest nitrogen content with compost-10 + PGPR, reflecting efficient uptake and translocation. Single Cross 131 responded moderately to compost-5 + PGPR, while Single Cross 132 showed reduced nitrogen levels at EC 6.3 dS/m, indicating stress sensitivity. The treatments, especially PGPR-based, enhanced nitrogen availability.

Proline and peroxidase, which are stress indicators, were strongly correlated with salinity (0.94 and 0.69 in 2023, 0.946 and 0.678 in 2024) but were minimally affected by the treatments. Single Cross 132 showed elevated levels, signaling high stress, while Single Cross 178 had lower levels with compost-10 + PGPR, indicating better stress management. Single Cross 131 displayed intermediate responses. The treatments mitigated osmotic and oxidative stress.

Chlorophyll content, negatively affected by salinity (−0.76 in 2023, −0.726 in 2024), improved slightly with the treatments (0.11 in 2023, 0.122 in 2024). Single Cross 178 retained higher chlorophyll with compost-10 + PGPR, supporting photosynthesis, while Single Cross 132 showed the lowest levels at EC_e_ 6.3 dS/m. Soil parameters, like available nitrogen, soil organic matter, and microbial populations, were enhanced by the treatments, particularly compost-10 + PGPR, which also reduced pH, EC, SAR, ESP, and bulk density, while increasing porosity. Single Cross 178 consistently thrived, Single Cross 131 showed moderate benefits, and Single Cross 132 was least resilient under high salinity.

## 3. Discussion

This study provides compelling evidence that integrated applications of compost and PGPR significantly enhance maize productivity and soil health under salinity stress, offering a sustainable strategy for agriculture in saline environments. The two-year field experiments conducted in 2023 and 2024 revealed substantial improvements in maize yield, nitrogen dynamics, physiological stress responses, and soil physicochemical and biological properties across three maize varieties under two salinity levels (EC_e_ 3.5 and 6.3 dS/m). These findings align with and extend the existing literature, highlighting the synergistic effects of organic amendments and microbial inoculants. The discussion below systematically compares our results with previously published studies, focusing on the effects of similar treatments on plant and soil properties and elucidating the mechanisms by which compost and PGPR mitigate salinity stress in maize cultivation.

Maize grain and straw yields were markedly improved by compost and PGPR treatments, with the most pronounced effects observed in the compost-10 + PGPR treatment, particularly under high salinity (EC_e_ 6.3 dS/m). Grain yields increased by up to 197%, and straw yields nearly tripled in Single Cross 178 compared to the control, demonstrating the efficacy of combined treatments. These results align closely with Hafez et al. [24], who reported a 75.2% increase in wheat grain yield with sugarcane bagasse and zinc oxide nanoparticles under salinity stress, attributed to improved nutrient retention and reduced oxidative stress. Similarly, Wang et al. [25] observed an 18.32% increase in maize grain yield with humic acid under saline-alkali conditions, linked to enhanced soil structure and nutrient availability. Our findings exceed the 76.1% seed yield increase reported by Alshaal et al. [8] with biochar and PGPR, suggesting that our compost formulations may provide superior organic matter and microbial synergy. The mechanisms likely involve enhanced nutrient uptake facilitated by PGPR, which produces indole-3-acetic acid (IAA) and solubilize phosphates, as noted by Viti et al. [26], who reported increased PGPR populations improving nutrient availability. Compost likely improved soil water retention and reduced sodium toxicity, enabling better root development and photosynthetic efficiency, as supported by El-Akhdar et al. [27], who observed a 50% increase in SOM with compost and PGPR. The superior performance of Single Cross 178 suggests genetic advantages in stress tolerance, possibly due to enhanced root architecture or ion selectivity, warranting further investigation.

The contents of N-grain and N-straw showed significant improvements with compost-10 + PGPR, reaching 1.36% and 2.24% at EC_e_ 3.5 dS/m, respectively, and maintaining high levels under EC_e_ 6.3 dS/m. Single Cross 178 exhibited the most notable increases, with N-straw rising by 157% under high salinity. These results are consistent with Shabaan et al. [28], who reported a 47% increase in maize-grain nitrogen content with PGPR inoculation under salinity stress, attributed to enhanced nitrogen mineralization and uptake via ACC deaminase activity. Similarly, Pereira et al. [29] found a 41% increase in nitrogen-use efficiency in maize under drought stress with PGPR co-inoculation, linked to nitrogen fixation and phytohormone production. Our results surpass the 33.9% increase in leaf nitrogen content reported by Abd El-Mageed et al. [30] in rice with *Bacillus* strains, likely due to the synergistic effect of compost providing organic nitrogen pools. The mechanisms likely include PGPR-mediated nitrogen fixation and solubilization, as described by Masters-Clark et al. [31], who noted enhanced nutrient uptake with phosphate-solubilizing Pseudomonas. Compost likely increased soil organic matter, creating a favorable environment for microbial nitrogen cycling, as supported by Elhawat et al. [32], who reported a 137% increase in total nitrogen with intercropping. The varietal differences, particularly Single Cross 178’s strong response, suggest genetic predispositions to efficient nitrogen assimilation under stress.

The proline content in maize leaves increased dramatically under EC_e_ 6.3 dS/m, reaching 37.37 μmol/g FW with compost-10 + PGPR in Single Cross 178, indicating its role as an osmoprotectant. This aligns with Chattaraj et al. [33], who reported increased proline production in maize with PGPR under drought stress, contributing to osmotic adjustment. Our results are higher than the 48% reduction in proline reported by Alshaal et al. [8] with biochar and PGPR, suggesting that our treatments may enhance stress-induced proline accumulation rather than alleviate it entirely. Shabaan et al. [28] also noted an improved osmotic balance with PGPR, supporting our findings. The mechanisms likely involve PGPR-induced upregulation of proline biosynthesis pathways, as noted by Netrusov et al. [34], who linked exopolysaccharide production to osmotic stress mitigation. Compost likely improved water retention, reducing cellular dehydration and triggering proline accumulation, as supported by Wang et al. [25], who observed enhanced soil water content with organic amendments. Single Cross 178’s high proline levels suggest a robust stress response, possibly due to genetic advantages in osmoprotectant synthesis.

Peroxidase activity increased significantly under EC_e_ 6.3 dS/m, reaching 180.95 μmol H_2_O_2_/min/g FW with compost-10 + PGPR in Single Cross 178, indicating a strong antioxidant defense. This is consistent with Alharbi et al. [10], who reported a 102% increase in peroxidase activity in wheat with PGPR and zinc oxide nanoparticles under salinity stress, linked to reduced oxidative damage. Our results exceed the 79% increase reported by Alshaal et al. [8], suggesting that our compost-PGPR combination may enhance enzymatic activity more effectively. Paul and Lade [35] also noted increased antioxidant enzyme activity with PGPR, supporting our findings. The mechanisms likely involve PGPR-induced expression of antioxidant genes, as described by Chattaraj et al. [33], who linked *Bacillus* strains to superoxide dismutase and catalase upregulation. Compost likely provided organic substrates that supported microbial activity, enhancing antioxidant production, as supported by El-Akhdar et al. [27], who reported increased microbial activity with compost. Single Cross 178’s superior response suggests genetic advantages in oxidative stress mitigation.

The total chlorophyll content was better preserved under salinity stress with compost-10 + PGPR, reaching 2.94 mg/g FW at EC_e_ 3.5 dS/m and 1.55 mg/g FW at EC_e_ 6.3 dS/m in Single Cross 178. This aligns with Abd El-Mageed et al. [30], who reported a 5.1% increase in chlorophyll fluorescence in rice with *Bacillus* strains, and Hafez et al. [36], who observed a 72% increase in canola chlorophyll with organic amendments. Our results are comparable to the 209.3% increase in soybean chlorophyll reported by Alharbi et al. [37] with biochar and PGPR, indicating robust photosynthetic protection. The mechanisms likely involve PGPR-mediated improvements in nutrient uptake (e.g., magnesium for chlorophyll synthesis), as noted by Zaib et al. [38], who reported enhanced nutrient assimilation with Pseudomonas. Compost likely reduced sodium toxicity, preserving chloroplast integrity, as supported by Omara et al. [39], who observed a 46.7% increase in chlorophyll a with compost and PGPR. Single Cross 178’s high chlorophyll retention suggests genetic resilience in photosynthetic apparatus under stress.

Soil pH was significantly reduced by compost-10 + PGPR, dropping from 8.45 to 7.70 under EC_e_ 6.3 dS/m, with the Single Cross 178 variety showing the greatest reduction. This aligns with El-Akhdar et al. [27], who reported a 31.8% reduction in electrical conductivity with compost and PGPR, indirectly suggesting pH moderation. Hafez et al. (2025) [24] also noted reduced soil pH with sugarcane bagasse, supporting our findings. The mechanisms likely involve compost’s organic acids buffering soil alkalinity, as described by Wang et al. [25], who linked humic acid to pH regulation. PGPR may enhance organic acid production, further lowering pH, as supported by Viti et al. [26]. The Single Cross 178 variety’s strong response suggests varietal differences in rhizosphere interactions with amendments.

The EC_e_ decreased significantly with compost-10 + PGPR, dropping from 7.67 to 2.30 dS/m under EC_e_ 6.3 dS/m, with the Single Cross 132 variety showing consistent reductions. This is consistent with Alshaal et al. [8], who reported a 31.7% reduction in EC_e_ with biochar and PGPR, and Omara et al. [39], who observed a 47% reduction in exchangeable sodium percentage. The mechanisms likely involve compost’s CEC, which binds sodium ions, and PGPR’s exopolysaccharide production, which reduces sodium availability, as noted by Netrusov et al. [34]. The Single Cross 132 variety 2’s response suggests favorable root exudates enhancing amendment efficacy.

The SAR and ESP were significantly reduced by compost-10 + PGPR, with the SAR dropping by 26.5% and ESP by 33.7% under EC_e_ 6.3 dS/m. This aligns with El-Akhdar et al. [27], who reported a 17.8% SAR reduction, and Hafez et al. [36], who observed a 31% ESP decrease. The mechanisms likely involve compost’s ability to improve soil structure and PGPR’s role in sodium sequestration, as supported by Shabaan et al. [28]. The Single Cross 178 variety 3’s strong ESP reduction suggests enhanced rhizosphere dynamics.

The soil bulk density decreased and the total porosity increased with compost-10 + PGPR, reaching 1.27 g/cm^3^ and 52.07% at EC_e_ 3.5 dS/m, respectively, with the Single Cross 132 variety showing the greatest improvements. This is consistent with Wang et al. [25], who reported a 60.52% increase in soil macro-aggregates, and Elhawat et al. [32], who noted an 11% increase in water retention. The mechanisms likely involve compost’s organic matter improving soil aggregation and PGPR’s root growth stimulation, as noted by Viti et al. [26]. The Single Cross 132 variety’s response suggests genetic advantages in root–soil interactions.

The SOM and Ava-N increased significantly with compost-10 + PGPR, reaching 1.28% and 40.63 ppm at EC_e_ 3.5 dS/m, respectively, with the Single Cross 132 variety showing the highest SOM and the Single Cross 131 variety the highest Ava-N. This aligns with El-Akhdar et al. [27], who reported a 50% SOM increase, and Shabaan et al. [28], who noted a 47% increase in grain nitrogen. The mechanisms involve compost’s organic inputs and PGPR’s nitrogen fixation, as supported by Masters-Clark et al. [31]. Varietal differences suggest genotype-specific microbial interactions.

The total bacterial and fungal populations were enhanced by compost-5 + PGPR, reaching 7.3 × 10^7^ and 2.0 × 10^5^ CFU/g at EC_e_ 3.5 dS/m, respectively, with the Single Cross 131 variety showing the highest bacterial counts and the Single Cross 178 variety showing the highest fungal counts. This is consistent with Viti et al. [26], who reported a 50% increase in fluorescent pseudomonads, and Alharbi et al. [9], who noted a 94.68% increase in microbial biomass. The mechanisms involve PGPR’s proliferation and compost’s nutrient provision, as noted by Xiang et al. [40]. Varietal differences suggest distinct rhizosphere microbiomes.

In conclusion, the integrated application of compost and PGPR significantly mitigates salinity stress in maize by enhancing yield, nitrogen uptake, physiological resilience, and soil health. The synergistic effects of organic matter and microbial activity provide a sustainable approach to saline agriculture, with varietal differences highlighting the importance of genotype selection. These findings advance the literature and offer practical solutions for farmers in saline regions.

## 4. Materials and Methods

### 4.1. Experimental Design and Setup

The field experiments were conducted at the Experimental Research Station Farm of the Agricultural Research Institute in Sakha, Kafr El-Sheikh Governorate, Egypt (31°06′57″ N, 30°56′30″ E) during the 2023 and 2024 growing seasons. Throughout the growing season, average daily temperatures vary from 25 to 33 °C (maximum) and 12 to 18 °C (minimum), with monthly precipitation of 0–20 mm, relative humidity between 46–77%, and sunshine hours ranging from 9 to 12 h per day. The study aimed to evaluate the effects of compost, PGPR, and their combination on three maize varieties under saline soil conditions in clay soil (Table 6).

Maize (*Zea mays* L.) seeds of three varieties (Single Cross 131, Single Cross 132, and Single Cross 178) were provided by the Field Crop Research Institute, Agricultural Research Center, Cereals Department, Sakha Agriculture Research Station, Kafr El-Sheikh, Egypt. The three maize varieties—Single Cross 131, 132, and 178—are high-yielding hybrids with plant heights of 2.5–3.0 m, green stalks post-harvest, and life cycles of 90–100 days. Single Cross 131 (11.3–13.6 t/ha) adapts well to irrigated conditions with 25–30 cm cobs. Single Cross 132 (11.3–14.0 t/ha) is drought-tolerant, produces two 20–25 cm cobs per plant, and suits soybean rotation. Single Cross 178 (12.7–14.5 t/ha) offers superior yield and lodging resistance. All varieties show strong pest and disease resistance, with grain yield purity of 78–80%. The experiment was designed as a split-block layout with four replications. The main plots consisted of two soil salinity levels (EC_e_ 3.5 and 6.3 dS/m) and the three maize varieties, while the subplots included four treatments: compost, PGPR, compost + PGPR, and a control (no compost or PGPR applied).

Maize seeds were sown at a rate of 25 kg/ha on 15 June 2023 and 11 June 2024, after wheat cultivation. The area of the experimental plot was 15 m^2^ (5 m × 3 m), including 4 rows, 5 m long, with row spacing of 0.75 m. Three seeds were planted per hill, with 0.25 m spacing between the hills and 0.75 m between the rows. Post-germination, the seedlings were thinned to one per hill. Weeds were manually controlled three times each season. Harvesting occurred on September 24 for both 2023 and 2024. Four plants from the central rows of each plot were randomly sampled for growth and yield assessments. For maize cultivation, fertilization followed the recommendations of the Egyptian Ministry of Agriculture and Land Reclamation. Nitrogen was applied at a rate of 288 kg N/ha using ammonium nitrate (33.5% N), split into two equal doses administered before the first and second irrigations following seed sowing. Phosphorus was supplied at 360 kg P_2_O₅/ha as calcium superphosphate (15.5% P_2_O₅), which was broadcast and incorporated into the soil during tillage. Potassium was applied at 120 kg K_2_O/ha using potassium sulfate (48% K_2_O), also broadcast and incorporated during soil tillage. Irrigation was applied eight times per season using fresh water (EC = 0.66 dS/m) when 50% of available soil water was depleted.

No pesticides were used during the cultivation process, ensuring an organic approach to crop management. Weed control was performed manually through regular hand-weeding techniques, promoting a labor-intensive but environmentally friendly method to maintain crop health. Throughout the growing period, careful monitoring revealed no visible signs or symptoms of fungal infections, indicating robust plant resilience and favorable environmental conditions.

The PGPR, *Azospirillum brasilense* SWERI 111, was supplied by the Microbiology Department, Soils, Water and Environment Research Institute, Agricultural Research Center, Sakha Agriculture Research Station, Kafr El-Sheikh, Egypt. The bacteria were cultured in Nutrient Broth (NB) liquid medium with the following composition (g/L): beef extract 1.0, peptone 5.0, yeast extract 2.0, and sodium chloride 3.0, adjusted to a pH of 6.8 ± 0.2 at 28 °C. Pure isolates were cultivated in 500 mL flasks containing 250 mL NB, incubated on a rotary shaker at 28 °C for 8 h daily. After three days, peat-based inoculants were prepared following the method of the authors of [41]. Cell suspensions with a concentration of 1×10^7^ CFU/mL were mixed with sterilized peat at a ratio of 50 mL NB per 100 g peat. The inoculated peat was thoroughly mixed and matured at room temperature for 48 h. Prior to sowing, maize seeds were coated with the PGPR peat inoculum at a rate of 950 g/ha, using a 10% Arabic gum water solution as an adhesive. Coated seeds were air-dried in the shade for 30 min.

The compost used as an organic fertilizer was provided by the Microbiology Department, Sakha Agricultural Research Station, Kafr El-Sheikh, Egypt. It comprised 70% plant materials and 30% animal waste, a ratio designed to optimize the carbon-to-nitrogen balance for effective decomposition. Compost was applied at rates of 5 and 10 t/ha during soil tillage to ensure uniform distribution. The physical and chemical properties of the compost are detailed in Table 7.

### 4.2. Plant and Soil Sampling Procedures

#### 4.2.1. Plant-Related Measurements

Eighty days after planting, the total chlorophyll content (chlorophyll a and b) was determined using spectrophotometry following the method outlined by the authors of [42]. Measurements were taken on the youngest fully expanded leaves, with six replicates per plot.

Proline, an important osmolyte and osmoprotectant, was quantified in leaves collected 80 days after sowing, following the method of the authors of [43]. Briefly, 0.5 g of the uppermost fully expanded leaves were homogenized in 3% sulfuric acid and centrifuged at 12,000× *g* for 5 min. The supernatant was reacted with ninhydrin reagent, mixed with toluene, and the absorbance was recorded at 520 nm using a UV-160A spectrophotometer (Shimadzu, Kyoto, Japan).

Peroxidase (POX: EC 1.11.1.7; µmol H_2_O_2_/g FW/min) activity was assessed using o-phenylenediamine as a chromogenic substrate in the presence of H_2_O_2_ and enzyme extract, with absorbance measured at 417 nm, as described by the authors of [44].

At harvest, 10 g of grains per treatment were air-dried, ground, and analyzed for nitrogen content using the micro Kjeldahl method [45]. Nitrogen content (mg/kg) in grains and straw was determined in finely ground samples digested with a 3:1 (*v*/*v*) HNO_3_:HClO_4_ mixture. Grain and straw yields were measured after harvesting the experimental plots.

#### 4.2.2. Soil-Related Measurements

At the experiment’s end, soil samples were collected from the 0–20 cm depth in triplicate from each plot. For chemical analysis, samples were air-dried, crushed, and passed through a 2 mm sieve. For biochemical analysis, additional triplicate samples were sieved through an 8 mm sieve after removing gravel, stones, and plant debris, and they were stored at −20 °C in polyethylene bags.

Soil pH was measured in a 1:2.5 soil-water suspension using a Jenway 3510 pH meter (Cole-Parmer, Westwood Ave, Long Branch, NJ, USA). Soil electrical conductivity (EC_e_) was determined in a soil paste extract using a Jenway 4310 EC meter (USA). The sodium adsorption ratio (SAR), a key indicator of soil sodicity affecting structure, permeability, and plant growth, was calculated based on sodium (Na^+^), calcium (Ca^2+^), and magnesium (Mg^2+^) ion concentrations (meq/L) in the soil paste extract, using the formula provided by the authors of [45].

$$SAR=\frac{\left[{Na}^{+}\right]}{\sqrt[{2}]{\frac{\left[{Ca}^{2+}\right]+[{Mg}^{2+}]}{2}}}$$

Soil organic matter (SOM) content was measured using the Walkley–Black chromic acid wet oxidation method with finely ground, air-dried soil samples (<0.25 mm) [45]. Available soil nitrogen (Ava-N) was extracted using 1 M potassium chloride and quantified via the Kjeldahl method [45]. The total bacterial count in soil was assessed at 80 days post-seed sowing as described in [41]. Fungal counts were determined using the plate method outlined in [41].

### 4.3. Statistical Analysis

Data were analyzed using analysis of variance (ANOVA). Data analysis was conducted utilizing Microsoft Excel 2010 (representing mean values with their respective standard deviations) and the SPSS 22.0 software package by SPSS Inc. based in Chicago, IL, USA. Post hoc analysis was then performed using Tukey’s test to distinguish between means, with statistical significance established at a significance level of *p* ≤ 0.05.

## 5. Conclusions

This study demonstrates that integrated compost and PGPR applications significantly enhance maize productivity and soil health under salinity stress, offering a sustainable solution for agriculture in saline environments. The compost-10 + PGPR treatment markedly improved grain and straw yields (up to 197% and 300%, respectively), nitrogen uptake, physiological stress responses, and soil properties, with Single Cross 178 showing superior tolerance. These findings highlight the synergistic effects of organic amendments and microbial inoculants in mitigating salinity-induced constraints through enhanced nutrient cycling, antioxidant defenses, and soil structure improvement. However, limitations include the study’s focus on only three maize varieties and two salinity levels, potentially limiting generalizability, and the lack of long-term data on treatment sustainability. Future research should explore a broader range of genotypes and salinity gradients, assess multi-year impacts, and investigate molecular mechanisms underlying varietal differences and PGPR-compost interactions. Additionally, scaling these interventions in diverse agroecological contexts and evaluating their economic feasibility could facilitate practical adoption, advancing global food security in saline-affected regions.

## Figures and Tables

**Table 1:** Effects of compost and PGPR treatments on grain yield, straw yield, and nitrogen content in three maize varieties grown under two salinity levels (EC_e_ 3.5 and 6.3 dS/m) during 2023 and 2024.

			Grain Yield (t/ha)		Straw Yield (t/ha)		N-Grain (%)		N-Straw (%)	
			2023	2024	2023	2024	2023	2024	2023	2024
Variety		131	6.29 ± 0.007 b	6.29 ± 0.007 b	14.60 ± 0.016 a	14.60 ± 0.015 a	1.11 ± 0.001 a	1.15 ± 0.001 a	1.66 ± 0.002 a	1.62 ± 0.002 a
		132	6.86 ± 0.007 b	6.86 ± 0.007 b	14.75 ± 0.015 a	14.75 ± 0.015 a	1.12 ± 0.001 a	1.15 ± 0.001 a	1.68 ± 0.002 a	1.64 ± 0.002 a
		178	7.58 ± 0.005 a	7.58 ± 0.005 a	13.94 ± 0.013 b	13.94 ± 0.013 b	1.12 ± 0.001 a	1.16 ± 0.001 a	1.67 ± 0.001 a	1.61 ± 0.001 a
Salinity		EC 3.5	7.58 ± 0.007	7.59 ± 0.007	15.54 ± 0.016	15.59 ± 0.016	1.16 ± 0.001	1.21 ± 0.001	1.83 ± 0.002	1.77 ± 0.002
		EC 6.3	6.85 ± 0.006	6.84 ± 0.006	13.98 ± 0.013	13.97 ± 0.013	1.10 ± 0.001	1.14 ± 0.001	1.62 ± 0.001	1.57 ± 0.001
Treatments		Control	3.57 ± 0.017 e	3.52 ± 0.016 e	9.11 ± 0.043 e	8.99 ± 0.042 e	0.93 ± 0.004 c	0.88 ± 0.004 d	1.00 ± 0.005 d	0.99 ± 0.005 d
		Compost-5	5.86 ± 0.003 d	5.86 ± 0.003 d	11.30 ± 0.006 d	11.30 ± 0.006 d	1.11 ± 0.001 b	1.21 ± 0.001 b	1.69 ± 0.001 b	1.63 ± 0.001 b
		Compost-10	7.33 ± 0.010 c	7.33 ± 0.010 c	13.84 ± 0.020 c	13.84 ± 0.020 c	1.19 ± 0.002 b	1.24 ± 0.002 b	1.75 ± 0.003 b	1.73 ± 0.002 b
		PGPR	5.47 ± 0.001 d	5.47 ± 0.001 d	10.45 ± 0.002 d	10.44 ± 0.002 d	0.97 ± 0.000 c	1.01 ± 0.000 c	1.34 ± 0.000 c	1.30 ± 0.000 c
		Compost-5 + PGPR	9.08 ± 0.003 b	9.11 ± 0.003 b	20.31 ± 0.007 b	20.38 ± 0.007 b	1.24 ± 0.000 a	1.28 ± 0.000 a	2.08 ± 0.001 a	1.97 ± 0.001 a
		Compost-10 + PGPR	10.16 ± 0.004 a	10.19 ± 0.004 a	21.58 ± 0.010 a	21.64 ± 0.010 a	1.26 ± 0.001 a	1.31 ± 0.001 a	2.15 ± 0.001 a	2.12 ± 0.001 a
Interaction										
Maize variety	Salinity (dS/m)	Treatments								
Single Cross 131	EC_e_ 3.5	Control	4.77 ± 0.022 x	4.76 ± 0.022 x	10.49 ± 0.002 v	10.47 ± 0.049 w	0.97 ± 0.005 r	0.91 ± 0.004 w	1.17 ± 0.005 x	1.11 ± 0.005 z
		Compost-5	6.42 ± 0.003 q	6.42 ± 0.003 q	12.11 ± 0.006 s	12.11 ± 0.006 s	1.11 ± 0.001 o	1.29 ± 0.001 g	1.95 ± 0.001 l	1.86 ± 0.001 l
		Compost-10	8.70 ± 0.015 j	8.70 ± 0.015 j	14.68 ± 0.009 l	14.68 ± 0.025 l	1.23 ± 0.002 e	1.31 ± 0.002 e	1.97 ± 0.003 k	1.91 ± 0.003 i
		PGPR	4.99 ± 0.001 w	4.99 ± 0.001 w	12.18 ± 0.064 r	12.18 ± 0.003 r	1.02 ± 0.000 p	1.05 ± 0.000 r	1.44 ± 0.000 s	1.48 ± 0.000 p
		Compost-5 + PGPR	8.86 ± 0.003 i	8.92 ± 0.003 h	21.48 ± 0.006 f	21.62 ± 0.007 f	1.26 ± 0.000 d	1.33 ± 0.000 c	2.17 ± 0.001 d	2.01 ± 0.001 e
		Compost-10 + PGPR	10.67 ± 0.005 d	10.72 ± 0.005 d	21.72 ± 0.021 e	21.83 ± 0.010 e	1.36 ± 0.001 a	1.34 ± 0.001 b	2.23 ± 0.001 b	2.24 ± 0.001 a
	EC_e_ 6.3	Control	2.93 ± 0.014 cc	2.85 ± 0.013 cc	8.46 ± 0.002 aa	8.22 ± 0.038 bb	0.88 ± 0.004 v	0.85 ± 0.004 y	0.79 ± 0.004 aa	0.83 ± 0.004 bb
		Compost-5	3.78 ± 0.002 z	3.78 ± 0.002 z	10.63 ± 0.008 u	10.62 ± 0.005 u	1.12 ± 0.001 n	1.13 ± 0.001 o	1.45 ± 0.001 r	1.41 ± 0.001 t
		Compost-10	5.46 ± 0.009 u	5.46 ± 0.009 u	14.05 ± 0.012 n	14.05 ± 0.024 n	1.15 ± 0.002 l	1.17 ± 0.002 m	1.53 ± 0.003 o	1.51 ± 0.003 o
		PGPR	3.06 ± 0.001 bb	3.06 ± 0.001 bb	10.15 ± 0.035 w	10.13 ± 0.002 x	0.92 ± 0.000 s	0.95 ± 0.000 s	1.20 ± 0.000 v	1.18 ± 0.000 v
		Compost-5 + PGPR	6.92 ± 0.002 n	6.92 ± 0.002 n	19.77 ± 0.006 g	19.77 ± 0.006 g	1.22 ± 0.000 gh	1.25 ± 0.000 j	1.99 ± 0.001 j	1.89 ± 0.001 j
		Compost-10 + PGPR	8.86 ± 0.004 i	8.86 ± 0.004 i	19.52 ± 0.024 h	19.52 ± 0.009 h	1.15 ± 0.001 kl	1.26 ± 0.001 i	2.03 ± 0.001 h	1.99 ± 0.001 f
Single Cross 132	EC_e_ 3.5	Control	4.48 ± 0.021 y	4.47 ± 0.021 y	9.79 ± 0.002 x	9.77 ± 0.046 y	0.99 ± 0.005 q	0.92 ± 0.004 u	1.19 ± 0.006 w	1.15 ± 0.005 x
		Compost-5	5.98 ± 0.003 s	5.98 ± 0.003 s	12.11 ± 0.006 s	12.11 ± 0.006 s	1.11 ± 0.001 o	1.30 ± 0.001 f	1.92 ± 0.001 m	1.81 ± 0.001 n
		Compost-10	9.46 ± 0.016 g	9.46 ± 0.016 f	12.60 ± 0.009 q	12.60 ± 0.021 q	1.23 ± 0.002 f	1.31 ± 0.002 e	2.00 ± 0.003 i	2.02 ± 0.003 d
		PGPR	5.12 ± 0.001 v	5.12 ± 0.001 v	10.84 ± 0.047 t	10.84 ± 0.002 t	1.03 ± 0.000 p	1.09 ± 0.000 q	1.49 ± 0.000 p	1.37 ± 0.000 u
		Compost-5 + PGPR	10.43 ± 0.003 f	10.50 ± 0.003 e	23.98 ± 0.005 c	24.15 ± 0.008 c	1.27 ± 0.000 c	1.32 ± 0.000 d	2.18 ± 0.001 c	2.08 ± 0.001 c
		Compost-10 + PGPR	11.14 ± 0.005 c	11.20 ± 0.005 c	25.12 ± 0.024 b	25.23 ± 0.012 b	1.34 ± 0.001 b	1.35 ± 0.001 a	2.23 ± 0.001 b	2.22 ± 0.001 b
	EC_e_ 6.3	Control	3.57 ± 0.017 aa	3.47 ± 0.016 aa	7.46 ± 0.002 cc	7.25 ± 0.034 dd	0.87 ± 0.004 v	0.85 ± 0.004 x	0.83 ± 0.004 y	0.84 ± 0.004 aa
		Compost-5	5.69 ± 0.003 t	5.68 ± 0.003 t	12.89 ± 0.007 p	12.89 ± 0.006 p	1.11 ± 0.001 o	1.12 ± 0.001 op	1.45 ± 0.001 r	1.44 ± 0.001 r
		Compost-10	6.49 ± 0.011 p	6.49 ± 0.011 p	14.36 ± 0.012 m	14.37 ± 0.024 m	1.15 ± 0.002 l	1.16 ± 0.002 n	1.53 ± 0.003 o	1.51 ± 0.003 o
		PGPR	4.78 ± 0.001 x	4.77 ± 0.001 x	9.53 ± 0.039 y	9.51 ± 0.002 z	0.91 ± 0.000 t	0.92 ± 0.000 uv	1.22 ± 0.000 u	1.17 ± 0.000 w
		Compost-5 + PGPR	6.95 ± 0.002 m	6.95 ± 0.002 m	18.57 ± 0.005 i	18.57 ± 0.006 i	1.23 ± 0.000 f	1.24 ± 0.000 k	2.00 ± 0.001 i	2.01 ± 0.001 e
		Compost-10 + PGPR	8.25 ± 0.004 l	8.26 ± 0.004 i	19.76 ± 0.000 g	19.76 ± 0.009 g	1.19 ± 0.001 i	1.26 ± 0.001 i	2.07 ± 0.001 g	2.08 ± 0.001 c
Single Cross 178	EC_e_ 3.5	Control	2.12 ± 0.010 dd	2.12 ± 0.010 dd	10.11 ± 0.000 w	10.09 ± 0.047 x	1.00 ± 0.005 q	0.91 ± 0.004 vw	1.19 ± 0.006 vw	1.14 ± 0.005 y
		Compost-5	6.44 ± 0.003 q	6.44 ± 0.003 q	10.55 ± 0.005 v	10.55 ± 0.005 v	1.11 ± 0.001 o	1.29 ± 0.001 g	1.95 ± 0.001 l	1.82 ± 0.001 m
		Compost-10	6.92 ± 0.012 n	6.92 ± 0.012 n	14.05 ± 0.005 n	14.05 ± 0.024 n	1.22 ± 0.002 fg	1.32 ± 0.002 d	1.96 ± 0.003 k	1.90 ± 0.003 j
		PGPR	6.33 ± 0.001 r	6.33 ± 0.001 r	10.86 ± 0.049 t	10.85 ± 0.002 t	1.03 ± 0.000 p	1.12 ± 0.000 b	1.48 ± 0.000 q	1.46 ± 0.000 q
		Compost-5 + PGPR	12.01 ± 0.004 a	12.09 ± 0.004 a	21.81 ± 0.006 d	21.95 ± 0.007 d	1.26 ± 0.000 c	1.33 ± 0.000 c	2.16 ± 0.001 e	1.98 ± 0.001 g
		Compost-10 + PGPR	11.52 ± 0.005 b	11.58 ± 0.005 b	25.34 ± 0.025 a	25.46 ± 0.012 a	1.35 ± 0.001 a	1.35 ± 0.001 a	2.24 ± 0.001 a	2.21 ± 0.001 b
	EC_e_ 6.3	Control	3.54 ± 0.017 aa	3.44 ± 0.016 aa	8.38 ± 0.003 bb	8.14 ± 0.038 cc	0.89 ± 0.004 u	0.83 ± 0.004 z	0.81 ± 0.004 z	0.83 ± 0.004 bb
		Compost-5	6.83 ± 0.003 o	6.83 ± 0.003 o	9.53 ± 0.007 y	9.53 ± 0.005 z	1.13 ± 0.001 m	1.12 ± 0.001 p	1.43 ± 0.001 t	1.42 ± 0.001 s
		Compost-10	6.93 ± 0.000 mn	6.93 ± 0.000 mn	13.29 ± 0.010 o	13.29 ± 0.000 o	1.16 ± 0.000 k	1.16 ± 0.000 n	1.54 ± 0.000 n	1.50 ± 0.000 o
		PGPR	8.56 ± 0.000 k	8.54 ± 0.000 k	9.14 ± 0.040 z	9.12 ± 0.000 aa	0.91 ± 0.000 t	0.94 ± 0.000 t	1.22 ± 0.000 u	1.15 ± 0.000 x
		Compost-5 + PGPR	9.28 ± 0.003 h	9.28 ± 0.003 g	16.25 ± 0.005 k	16.25 ± 0.005 k	1.22 ± 0.000 h	1.23 ± 0.000 l	2.00 ± 0.001 i	1.87 ± 0.001 k
		Compost-10 + PGPR	10.49 ± 0.003 e	10.49 ± 0.003 e	18.03 ± 0.024 j	18.04 ± 0.005 j	1.18 ± 0.000 j	1.27 ± 0.000 h	2.09 ± 0.001 f	1.97 ± 0.001 h

Means followed by different letters are significant according to Tukey’s test at level of *p* ≤ 0.05. Note, double letter is different from single letter.

**Table 2:** Effects of compost and PGPR treatments on proline content, peroxidase activity, and chlorophyll levels in maize under salinity stress (2023–2024).

			Proline Concentration (µmol/g FW)		Peroxidase Activity (µmol H_2_O_2_/min/g FW)		Total Chlorophyll (mg/g FW)	
			2023	2024	2023	2024	2023	2024
Variety		131	19.62 ± 0.46 a	19.42 ± 0.45 a	103.82 ± 1.53 a	101.83 ± 1.49 a	1.84 ± 0.06 a	2.07 ± 0.11 a
		132	15.28 ± 0.29 b	15.21 ± 0.44 b	45.05 ± 0.41 b	44.29 ± 0.67 b	1.50 ± 0.05 a	1.62 ± 0.04 a
		178	11.70 ± 0.29 c	11.71 ± 0.32 c	30.30 ± 0.30 b	28.63 ± 2.30 c	1.11 ± 0.05 b	1.19 ± 0.07 b
Salinity		EC 3.5	3.06 ± 0.19	3.09 ± 0.22	23.50 ± 0.28	23.02 ± 0.37	1.85 ± 0.06	2.04 ± 0.09
		EC 6.3	19.73 ± 0.41	19.63 ± 0.47	78.43 ± 1.04	76.26 ± 2.42	1.34 ± 0.04	1.44 ± 0.06
Treatments		Control	14.29 ± 0.40 c	14.13 ± 0.55 c	55.67 ± 0.80 d	54.80 ± 0.77 d	1.38 ± 0.04 c	1.47 ± 0.09 c
		Compost-5	15.21 ± 0.35 b	15.00 ± 0.35 b	57.93 ± 0.37 c	57.02 ± 0.55 c	1.46 ± 0.07 b	1.58 ± 0.07 b
		Compost-10	15.69 ± 0.22 a	15.49 ± 0.21 b	59.30 ± 0.45 b	58.38 ± 0.45 b	1.49 ± 0.07 b	1.70 ± 0.12 a
		PGPR	15.78 ± 0.35 a	15.77 ± 0.30 a	61.61 ± 1.72 a	60.61 ± 1.45 a	1.49 ± 0.05 b	1.62 ± 0.05 b
		Compost-5 + PGPR	15.99 ± 0.36 a	16.00 ± 0.54 a	61.09 ± 0.59 b	57.46 ± 4.80 b	1.54 ± 0.03 a	1.69 ± 0.06 a
		Compost-10 + PGPR	16.22 ± 0.41 a	16.28 ± 0.46 a	62.72 ± 0.54 a	61.24 ± 0.91 a	1.54 ± 0.04 a	1.71 ± 0.05 a
Interaction								
Maize variety	Salinity (dS/m)	Treatments						
Single Cross 131	EC_e_ 3.5	Control	3.57 ± 0.24 hijk	3.54 ± 0.28 ijk	35.05 ± 0.20 i	34.24 ± 1.50 ef	2.25 ± 0.06 a	2.47 ± 0.06 bc
		Compost-5	3.84 ± 0.39 hij	3.96 ± 0.14 ij	37.73 ± 1.41 i	37.20 ± 0.19 ef	2.34 ± 0.09 a	2.59 ± 0.07 ab
		Compost-10	3.56 ± 0.11 hijk	3.58 ± 0.17 ijk	38.28 ± 1.51 i	37.32 ± 0.12 ef	2.28 ± 0.15 a	2.94 ± 0.52 a
		PGPR	3.55 ± 0.28 hijk	3.58 ± 0.34 ijk	38.82 ± 0.28 i	38.06 ± 0.17 ef	2.26 ± 0.12 a	2.48 ± 0.12 bc
		Compost-5 + PGPR	4.23 ± 0.70 h	4.18 ± 0.62 i	39.42 ± 0.07 i	38.25 ± 0.12 ef	2.39 ± 0.05 a	2.69 ± 0.17 ab
		Compost-10 + PGPR	3.91 ± 0.45 hi	3.92 ± 0.44 ij	39.18 ± 0.03 i	38.22 ± 0.94 ef	2.41 ± 0.12 a	2.70 ± 0.17 ab
	EC_e_ 6.3	Control	31.88 ± 0.50 c	31.34 ± 0.94 c	156.34 ± 0.21 d	153.97 ± 0.57 c	1.23 ± 0.01 cdef	1.40 ± 0.05 hijk
		Compost-5	34.57 ± 1.16 b	33.76 ± 1.07 b	163.39 ± 0.08 c	160.87 ± 1.37 bc	1.35 ± 0.02 cdef	1.47 ± 0.05 hij
		Compost-10	36.10 ± 0.28 a	35.54 ± 0.29 a	166.62 ± 0.24 bc	163.83 ± 1.62 abc	1.39 ± 0.03 cd	1.50 ± 0.06 ghij
		PGPR	36.33 ± 0.44 a	36.12 ± 0.24 a	178.84 ± 1.45 a	175.13 ± 7.06 a	1.37 ± 0.06 cde	1.47 ± 0.04 hij
		Compost-5 + PGPR	36.55 ± 0.38 a	36.44 ± 0.61 a	171.17 ± 0.33 b	168.30 ± 1.73 ab	1.41 ± 0.02 c	1.54 ± 0.02 efghi
		Compost-10 + PGPR	37.37 ± 0.63 a	37.13 ± 0.26 a	180.95 ± 0.06 a	176.57 ± 2.47 a	1.42 ± 0.02 c	1.55 ± 0.02 efghi
Single Cross 132	EC_e_ 3.5	Control	2.62 ± 0.17 ijk	2.59 ± 0.45 ijk	17.50 ± 0.56 jklm	17.40 ± 0.17 h	1.74 ± 0.06 b	1.91 ± 0.03 defg
		Compost-5	2.72 ± 0.13 ijk	2.74 ± 0.19 ijk	18.25 ± 1.13 jklm	18.02 ± 0.29 h	1.86 ± 0.11 b	2.00 ± 0.10 d
		Compost-10	2.80 ± 0.10 ijk	2.88 ± 0.10 ijk	18.95 ± 0.45 jkl	18.66 ± 0.47 h	1.84 ± 0.06 b	1.92 ± 0.06 def
		PGPR	2.82 ± 0.04 hijk	2.79 ± 0.04 ijk	19.08 ± 0.11 jk	18.63 ± 0.71 h	1.83 ± 0.02 b	1.94 ± 0.03 de
		Compost-5 + PGPR	3.18 ± 0.26 hijk	3.18 ± 0.27 ijk	19.28 ± 0.01 jk	18.88 ± 0.50 gh	1.86 ± 0.04 b	1.95 ± 0.03 de
		Compost-10 + PGPR	3.13 ± 0.16 hijk	3.54 ± 0.49 ijk	19.55 ± 0.05 j	19.17 ± 0.26 gh	1.92 ± 0.03 b	2.11 ± 0.00 cd
	EC_e_ 6.3	Control	26.40 ± 0.84 e	26.38 ± 0.93 e	67.80 ± 0.17 g	66.87 ± 1.50 d	0.98 ± 0.01 gh	1.10 ± 0.01 jklmn
		Compost-5	27.24 ± 0.24 de	26.83 ± 0.53 de	68.97 ± 0.34 fg	67.96 ± 0.43 d	1.08 ± 0.06 fg	1.12 ± 0.11 jklmn
		Compost-10	27.72 ± 0.28 de	27.51 ± 0.19 de	71.40 ± 0.19 efg	70.32 ± 0.02 d	1.21 ± 0.09 def	1.38 ± 0.04 hijkl
		PGPR	28.07 ± 0.63 d	27.83 ± 0.57 de	71.84 ± 0.58 efg	71.53 ± 0.49 d	1.18 ± 0.04 ef	1.31 ± 0.03 hijklm
		Compost-5 + PGPR	28.25 ± 0.15 d	27.95 ± 0.89 de	74.32 ± 0.46 e	72.23 ± 0.67 d	1.27 ± 0.03 cdef	1.38 ± 0.06 hijkl
		Compost-10 + PGPR	28.37 ± 0.48 d	28.27 ± 0.62 d	73.63 ± 0.42 ef	71.80 ± 0.57 d	1.24 ± 0.02 cdef	1.33 ± 0.02 hijklm
Single Cross 178	EC_e_ 3.5	Control	2.25 ± 0.12 k	2.26 ± 0.12 k	12.32 ± 0.61 n	12.18 ± 0.06 h	1.36 ± 0.06 cde	1.42 ± 0.02 hij
		Compost-5	2.45 ± 0.10 jk	2.40 ± 0.16 jk	13.21 ± 0.50 mn	12.75 ± 0.45 h	1.37 ± 0.05 cde	1.43 ± 0.03 hij
		Compost-10	2.55 ± 0.05 ijk	2.49 ± 0.04 jk	13.50 ± 0.17 mn	13.59 ± 0.13 h	1.41 ± 0.07 cd	1.52 ± 0.02 fghi
		PGPR	2.61 ± 0.03 ijk	2.62 ± 0.03 ijk	13.92 ± 1.54 lmn	13.33 ± 0.19 h	1.40 ± 0.03 cd	1.52 ± 0.04 fghi
		Compost-5 + PGPR	2.61 ± 0.08 ijk	2.65 ± 0.05 ijk	14.32 ± 0.35 klmn	14.06 ± 0.29 h	1.43 ± 0.02 c	1.56 ± 0.03 efgh
		Compost-10 + PGPR	2.73 ± 0.04 ijk	2.70 ± 0.02 ijk	14.60 ± 0.06 jklmn	14.43 ± 0.09 h	1.42 ± 0.03 c	1.58 ± 0.08 efgh
	EC_e_ 6.3	Control	19.05 ± 0.54 j	18.66 ± 0.58 h	45.03 ± 0.55 h	44.10 ± 0.79 ef	0.71 ± 0.06 i	0.51 ± 0.07 eo
		Compost-5	20.40 ± 0.06 fg	20.33 ± 0.01 g	46.03 ± 0.11 h	45.32 ± 0.55 ef	0.77 ± 0.10 i	0.86 ± 0.08 no
		Compost-10	21.44 ± 0.47 f	20.92 ± 0.51 fg	47.07 ± 0.67 h	46.55 ± 0.33 ef	0.84 ± 0.03 hi	0.93 ± 0.01 mno
		PGPR	21.32 ± 0.68 f	21.69 ± 0.61 fg	47.18 ± 0.87 h	46.99 ± 0.11 ef	0.88 ± 0.06 ghi	0.97 ± 0.05 lmn
		Compost-5 + PGPR	21.15 ± 0.62 f	21.62 ± 0.79 fg	48.03 ± 0.99 h	33.01 ± 0.50 fg	0.88 ± 0.03 ghi	1.00 ± 0.06 klmn
		Compost-10 + PGPR	21.81 ± 0.70 f	22.14 ± 0.95 f	48.40 ± 2.06 h	47.25 ± 0.12 e	0.86 ± 0.03 hi	0.97 ± 0.02 lmn

Means followed by different letters are significant according to Tukey’s test at level of *p* ≤ 0.05. Note, double letter is different from single letter.

**Table 3:** Changes in soil pH, electrical conductivity (EC_e_), and sodium adsorption ratio (SAR) under different treatments and salinity levels (2023–2024).

			pH		EC_e_		SAR	
			2023	2024	2023	2024	2023	2024
Variety		131	7.90 ± 0.01 a	8.09 ± 0.01 a	3.74 ± 0.01 c	3.87 ± 0.01 c	9.83 ± 0.37 c	10.00 ± 0.38 c
		132	7.61 ± 0.01 b	7.75 ± 0.01 b	4.09 ± 0.01 b	4.21 ± 0.01 b	10.31 ± 0.40 b	10.45 ± 0.41 b
		178	7.77 ± 0.01 a	7.87 ± 0.01 b	4.57 ± 0.01 a	4.70 ± 0.01 a	10.88 ± 0.42 a	11.03 ± 0.43 a
Salinity		EC 3.5	7.55 ± 0.01	7.60 ± 0.01	3.56 ± 0.01	3.63 ± 0.01	9.78 ± 0.45	9.87 ± 0.45
		EC 6.3	7.81 ± 0.01	8.01 ± 0.01	4.36 ± 0.01	4.50 ± 0.01	10.55 ± 0.38	10.71 ± 0.39
Treatments		Control	7.96 ± 0.04 a	8.08 ± 0.04 a	5.94 ± 0.03 a	6.08 ± 0.03 a	12.42 ± 0.52 a	12.57 ± 0.53 a
		Compost-5	7.80 ± 0.00 a	7.89 ± 0.00 b	4.05 ± 0.00 c	4.15 ± 0.00 c	10.24 ± 0.43 c	10.38 ± 0.43 c
		Compost-10	7.72 ± 0.01 b	7.87 ± 0.01 b	3.57 ± 0.00 d	3.58 ± 0.00 d	9.50 ± 0.37 d	9.49 ± 0.37 d
		PGPR	7.80 ± 0.00 a	8.10 ± 0.00 a	4.96 ± 0.00 b	5.19 ± 0.00 b	11.15 ± 0.44 b	11.41 ± 0.45 b
		Compost-5 + PGPR	7.66 ± 0.00 c	7.78 ± 0.00 c	3.27 ± 0.00 d	3.41 ± 0.00 d	9.59 ± 0.33 d	9.78 ± 0.33 d
		Compost-10 + PGPR	7.62 ± 0.00 c	7.71 ± 0.00 c	3.02 ± 0.00 e	3.15 ± 0.00 e	9.14 ± 0.30 e	9.32 ± 0.31 e
Interaction								
Maize variety	Salinity (dS/m)	Treatments						
Single Cross 131	EC_e_ 3.5	Control	7.92 ± 0.01 i	7.90 ± 0.00 j	4.10 ± 0.02 lm	4.26 ± 0.02 o	10.14 ± 0.66 ghiklm	10.33 ± 0.67 fghijk
		Compost-5	7.80 ± 0.04 j	7.80 ± 0.01 k	2.43 ± 0.00 w	2.76 ± 0.00 x	7.81 ± 0.52 p	8.32 ± 0.56 mn
		Compost-10	7.70 ± 0.01 k	7.70 ± 0.00 lm	2.76 ± 0.00 u	2.59 ± 0.00 z	8.72 ± 0.20 mnop	8.45 ± 0.19 mn
		PGPR	7.80 ± 0.00 j	7.80 ± 0.00 k	3.19 ± 0.00 s	3.44 ± 0.00 v	9.38 ± 0.21 klmno	9.73 ± 0.22 ijklm
		Compost-5 + PGPR	7.60 ± 0.00 l	7.65 ± 0.00 m	2.73 ± 0.00 uv	2.75 ± 0.00 y	9.12 ± 0.44 lmnop	9.15 ± 0.44 klmn
		Compost-10 + PGPR	7.70 ± 0.00 k	7.74 ± 0.04 l	2.30 ± 0.00 x	2.31 ± 0.00 aa	8.37 ± 0.40 nop	8.39 ± 0.40 mn
	EC_e_ 6.3	Control	8.22 ± 0.04 a	8.45 ± 0.00 c	6.91 ± 0.03 b	7.10 ± 0.03 b	13.71 ± 0.29 ab	13.90 ± 0.29 ab
		Compost-5	8.10 ± 0.00 cd	8.10 ± 0.01 h	4.39 ± 0.00 j	4.38 ± 0.00 l	10.93 ± 0.24 efghij	10.92 ± 0.23 efghij
		Compost-10	8.00 ± 0.01 gh	8.51 ± 0.00 b	3.24 ± 0.01 r	3.23 ± 0.01 w	8.72 ± 0.47 mnop	8.71 ± 0.84 lmn
		PGPR	8.00 ± 0.00 gh	8.46 ± 0.00 bc	5.28 ± 0.00 g	5.58 ± 0.00 g	11.14 ± 0.61 defgh	11.45 ± 0.63 defg
		Compost-5 + PGPR	8.00 ± 0.00 fgh	8.62 ± 0.00 a	3.72 ± 0.00 o	4.01 ± 0.00 q	9.88 ± 0.20 hijklm	10.25 ± 0.20 fghijk
		Compost-10 + PGPR	7.90 ± 0.00 i	8.33 ± 0.04 de	3.87 ± 0.00 n	4.08 ± 0.00 p	10.07 ± 0.20 ghijkl	10.34 ± 0.21 fghijk
Single Cross 132	EC_e_ 3.5	Control	7.52 ± 0.04 m	7.66 ± 0.00 m	5.19 ± 0.02 h	5.29 ± 0.02 i	11.41 ± 0.77 defg	11.52 ± 0.79 def
		Compost-5	7.40 ± 0.00 n	7.40 ± 0.01 o	3.72 ± 0.00 o	3.72 ± 0.00 s	9.66 ± 0.64 ijklmn	9.66 ± 0.46 jklm
		Compost-10	7.30 ± 0.01 o	7.30 ± 0.00 q	3.25 ± 0.01 r	3.23 ± 0.01 v	9.46 ± 0.21 klmno	9.44 ± 0.22 klmn
		PGPR	7.40 ± 0.00 n	7.68 ± 0.00 m	4.17 ± 0.00 k	4.33 ± 0.00 m	10.72 ± 0.24 fghijk	10.92 ± 0.26 efghij
		Compost-5 + PGPR	7.30 ± 0.00 o	7.35 ± 0.00 op	2.92 ± 0.00 t	2.94 ± 0.00 w	9.43 ± 0.46 klmno	9.46 ± 0.46 jklmn
		Compost-10 + PGPR	7.30 ± 0.00 o	7.33 ± 0.04 pq	2.14 ± 0.00 y	2.15 ± 0.00 bb	8.07 ± 0.39 op	8.09 ± 0.39 n
	EC_e_ 6.3	Control	8.12 ± 0.04 bc	8.26 ± 0.00 f	6.22 ± 0.03 d	6.32 ± 0.03 d	13.00 ± 0.27 abc	13.11 ± 0.28 abc
		Compost-5	7.90 ± 0.00 i	8.20 ± 0.01 g	4.40 ± 0.00 j	4.57 ± 0.00 j	10.94 ± 0.24 efghij	11.14 ± 0.24 defghi
		Compost-10	7.80 ± 0.01 j	8.17 ± 0.00 g	4.50 ± 0.01 i	4.50 ± 0.01 k	10.28 ± 0.56 ghijkl	10.28 ± 0.57 fghijk
		PGPR	7.90 ± 0.00 i	8.27 ± 0.00 ef	5.20 ± 0.00 h	5.45 ± 0.00 h	11.05 ± 0.61 defghi	11.31 ± 0.62 defgh
		Compost-5 + PGPR	7.70 ± 0.00 k	7.70 ± 0.00 lm	3.50 ± 0.00 q	3.76 ± 0.00 r	9.58 ± 0.19 jklmn	9.93 ± 0.20 hijkl
		Compost-10 + PGPR	7.70 ± 0.00 k	7.70 ± 0.00 lm	3.90 ± 0.00 n	4.21 ± 0.00 o	10.11 ± 0.20 ghijklmn	10.50 ± 0.21 fghijk
Single Cross 178	EC_e_ 3.5	Control	7.84 ± 0.04 j	7.82 ± 0.04 k	5.71 ± 0.03 e	5.82 ± 0.03 e	11.97 ± 0.81 cdef	12.09 ± 0.83 cde
		Compost-5	7.51 ± 0.00 m	7.51 ± 0.00 n	4.06 ± 0.00 m	4.07 ± 0.00 q	10.09 ± 0.67 ghijkl	10.11 ± 0.67 fghijkl
		Compost-10	7.49 ± 0.01 m	7.49 ± 0.01 n	3.60 ± 0.01 b	3.64 ± 0.01 s	9.96 ± 0.22 hijklm	10.02 ± 0.23 ghijkl
		PGPR	7.63 ± 0.00 l	7.92 ± 0.00 ij	5.60 ± 0.00 f	5.72 ± 0.00 f	12.43 ± 0.28 bcd	12.56 ± 0.28 bcd
		Compost-5 + PGPR	7.34 ± 0.00 o	7.39 ± 0.00 o	3.50 ± 0.00 q	3.52 ± 0.00 t	10.32 ± 0.50 ghijkl	10.35 ± 0.50 fghijk
		Compost-10 + PGPR	7.33 ± 0.00 o	7.36 ± 0.00 op	2.70 ± 0.00 v	2.72 ± 0.00 yz	9.06 ± 0.44 lmnop	9.10 ± 0.44 klmn
	EC_e_ 6.3	Control	8.17 ± 0.04 ab	8.37 ± 0.04 d	7.52 ± 0.04 h	7.67 ± 0.04 a	14.30 ± 0.30 a	14.44 ± 0.31 a
		Compost-5	8.05 ± 0.00 def	8.30 ± 0.00 ef	5.30 ± 0.00 g	5.41 ± 0.00 h	12.01 ± 0.26 cdef	12.13 ± 0.26 cde
		Compost-10	8.05 ± 0.00 efg	8.05 ± 0.00 h	4.10 ± 0.00 l	4.27 ± 0.00 n	9.82 ± 0.54 hijklm	10.02 ± 0.55 ghijkl
		PGPR	8.07 ± 0.00 cde	8.45 ± 0.00 c	6.30 ± 0.00 c	6.65 ± 0.00 c	12.17 ± 0.67 cde	12.50 ± 0.69 bcd
		Compost-5 + PGPR	7.98 ± 0.00 h	7.96 ± 0.00 i	3.24 ± 0.00 r	3.47 ± 0.00 u	9.22 ± 0.18 lmnop	9.54 ± 0.19 jklmn
		Compost-10 + PGPR	7.82 ± 0.00 j	7.81 ± 0.00 k	3.20 ± 0.00 s	3.45 ± 0.00 v	9.16 ± 0.18 lmnop	9.51 ± 0.19 jklmn

Means followed by different letters are significant according to Tukey’s test at level of *p* ≤ 0.05. Note, double letter is different from single letter.

**Table 4:** Changes in soil exchangeable sodium percentage (ESP), soil bulk density, and total porosity under different treatments and salinity levels (2023–2024).

			ESP		Bulk Density		Total Porosity	
			2023	2024	2023	2024	2023	2024
Variety		131	11.66 ± 0.43 b	11.84 ± 0.43 c	1.35 ± 0.00 a	1.36 ± 0.00 a	49.00 ± 0.07 a	48.65 ± 0.07 a
		132	12.22 ± 0.45 a	12.37 ± 0.46 b	1.35 ± 0.00 a	1.36 ± 0.00 a	49.03 ± 0.07 a	48.65 ± 0.07 a
		178	12.84 ± 0.47 a	13.01 ± 0.48 a	1.35 ± 0.00 a	1.36 ± 0.00 a	48.99 ± 0.06 a	48.65 ± 0.06 a
Salinity		EC 3.5	11.61 ± 0.52	11.71 ± 0.52	1.33 ± 0.00	1.33 ± 0.00	49.74 ± 0.07	49.76 ± 0.07
		EC 6.3	12.47 ± 0.43	12.65 ± 0.44	1.36 ± 0.00	1.37 ± 0.00	48.86 ± 0.06	48.38 ± 0.06
Treatments		Control	14.55 ± 0.56 a	14.70 ± 0.58 a	1.39 ± 0.01 a	1.43 ± 0.01 a	47.42 ± 0.25 d	45.97 ± 0.25 e
		Compost-5	12.13 ± 0.50 c	12.30 ± 0.50 c	1.35 ± 0.00 b	1.37 ± 0.00 b	48.91 ± 0.03 c	48.47 ± 0.03 c
		Compost-10	11.30 ± 0.42 d	11.29 ± 0.43 d	1.35 ± 0.00 b	1.34 ± 0.00 b	49.19 ± 0.07 b	49.31 ± 0.07 b
		PGPR	13.17 ± 0.48 b	13.46 ± 0.49 b	1.37 ± 0.00 a	1.40 ± 0.00 a	48.36 ± 0.01 c	47.17 ± 0.01 d
		Compost-5 + PGPR	11.41 ± 0.38 d	11.63 ± 0.38 d	1.33 ± 0.00 b	1.32 ± 0.00 c	49.73 ± 0.02 b	50.29 ± 0.02 a
		Compost-10 + PGPR	10.88 ± 0.36 e	11.09 ± 0.36 d	1.31 ± 0.00 c	1.31 ± 0.00 c	50.44 ± 0.02 a	50.69 ± 0.02 a
Interaction								
Maize variety	Salinity (dS/m)	Treatments						
Single Cross 131	EC_e_ 3.5	Control	12.04 ± 0.34 fghijk	12.26 ± 0.76 fghij	1.37 ± 0.01 de	1.44 ± 0.01 b	48.17 ± 0.03 lm	45.52 ± 0.25 n
		Compost-5	9.30 ± 0.75 o	9.92 ± 0.67 no	1.35 ± 0.00 j	1.34 ± 0.00 j	49.03 ± 0.09 gh	49.41 ± 0.03 g
		Compost-10	10.40 ± 0.64 lmno	10.08 ± 0.23 mno	1.34 ± 0.00 lm	1.33 ± 0.00 k	49.45 ± 0.01 ef	49.83 ± 0.08 f
		PGPR	11.17 ± 0.24 jklmn	11.58 ± 0.25 ijklm	1.36 ± 0.00 hi	1.36 ± 0.00 hi	48.68 ± 0.02 i	48.68 ± 0.01 h
		Compost-5 + PGPR	10.86 ± 0.25 klmno	10.90 ± 0.52 klmno	1.31 ± 0.00 n	1.28 ± 0.00 no	50.55 ± 0.02 c	51.68 ± 0.02 b
		Compost-10 + PGPR	9.97 ± 0.52 mno	10.00 ± 0.49 mno	1.30 ± 0.00 o	1.28 ± 0.00 o	50.94 ± 0.25 b	51.70 ± 0.02 b
	EC_e_ 6.3	Control	15.93 ± 0.48 ab	16.13 ± 0.31 ab	1.42 ± 0.01 a	1.45 ± 0.01 a	46.28 ± 0.03 p	45.15 ± 0.26 o
		Compost-5	12.94 ± 0.30 efghi	12.92 ± 0.26 efghi	1.36 ± 0.00 hi	1.37 ± 0.00 f	48.66 ± 0.09 ij	48.28 ± 0.03 j
		Compost-10	10.40 ± 0.27 lmno	10.38 ± 0.57 lmno	1.36 ± 0.00 ij	1.36 ± 0.00 i	48.69 ± 0.01 hi	48.69 ± 0.09 h
		PGPR	13.17 ± 0.56 defg	13.52 ± 0.69 defg	1.37 ± 0.00 efg	1.44 ± 0.00 b	48.30 ± 0.02 kl	45.66 ± 0.01 n
		Compost-5 + PGPR	11.74 ± 0.68 ghijkl	12.17 ± 0.23 fghij	1.34 ± 0.00 kl	1.34 ± 0.00 j	49.41 ± 0.02 f	49.41 ± 0.02 g
		Compost-10 + PGPR	11.97 ± 0.23 fghijkl	12.27 ± 0.24 fghij	1.33 ± 0.00 m	1.33 ± 0.00 k	49.81 ± 0.24 d	49.81 ± 0.02 f
Single Cross 132	EC_e_ 3.5	Control	13.47 ± 0.23 def	13.59 ± 0.87 def	1.36 ± 0.01 fghi	1.40 ± 0.01 d	48.55 ± 0.02 ijk	47.04 ± 0.25 l
		Compost-5	11.49 ± 0.86 hijklm	11.49 ± 0.75 jklmn	1.33 ± 0.00 m	1.33 ± 0.00 k	49.79 ± 0.08 de	49.79 ± 0.02 f
		Compost-10	11.27 ± 0.75 jklm	11.24 ± 0.25 klmno	1.32 ± 0.00 n	1.31 ± 0.00 m	50.20 ± 0.01 c	50.58 ± 0.08 d
		PGPR	12.71 ± 0.24 efghij	12.93 ± 0.27 efghi	1.35 ± 0.00 jk	1.37 ± 0.00 fg	49.06 ± 0.02 g	48.30 ± 0.01 j
		Compost-5 + PGPR	11.22 ± 0.27 jklmn	11.26 ± 0.53 klmno	1.31 ± 0.00 n	1.28 ± 0.00 no	50.55 ± 0.02 c	51.68 ± 0.02 b
		Compost-10 + PGPR	9.62 ± 0.53 no	9.64 ± 0.48 o	1.29 ± 0.00 p	1.27 ± 0.00 p	51.32 ± 0.25 a	52.07 ± 0.02 a
	EC_e_ 6.3	Control	15.19 ± 0.47 abc	15.31 ± 0.29 abc	1.41 ± 0.01 b	1.45 ± 0.01 a	46.66 ± 0.03 o	45.15 ± 0.26 o
		Compost-5	12.95 ± 0.29 defghi	13.18 ± 0.27 defghi	1.38 ± 0.00 d	1.41 ± 0.00 d	47.90 ± 0.09 m	46.77 ± 0.03 l
		Compost-10	12.21 ± 0.27 fghijk	12.21 ± 0.64 fghij	1.37 ± 0.00 efgh	1.37 ± 0.00 fgh	48.32 ± 0.01 jkl	48.32 ± 0.09 ij
		PGPR	13.07 ± 0.63 defgh	13.36 ± 0.69 defgh	1.39 ± 0.00 c	1.43 ± 0.00 c	47.55 ± 0.02 n	46.04 ± 0.01 m
		Compost-5 + PGPR	11.40 ± 0.68 ijklm	11.80 ± 0.23 hijkl	1.36 ± 0.00 ghi	1.36 ± 0.00 ghi	48.66 ± 0.02 ij	48.66 ± 0.02 hi
		Compost-10 + PGPR	12.01 ± 0.22 fghijkl	12.46 ± 0.24 fghij	1.33 ± 0.00 m	1.34 ± 0.00 j	49.81 ± 0.24 d	49.43 ± 0.02 g
Single Cross 178	EC_e_ 3.5	Control	14.08 ± 0.23 cde	14.21 ± 0.90 cde	1.37 ± 0.01 de	1.38 ± 0.01 e	48.17 ± 0.02 lm	47.79 ± 0.24 k
		Compost-5	11.99 ± 0.89 fghijkl	12.00 ± 0.77 fghijk	1.33 ± 0.00 m	1.33 ± 0.00 k	49.79 ± 0.08 de	49.79 ± 0.02 f
		Compost-10	11.84 ± 0.77 fghijkl	11.91 ± 0.26 ghijk	1.32 ± 0.00 n	1.32 ± 0.00 l	50.20 ± 0.08 c	50.20 ± 0.08 e
		PGPR	14.58 ± 0.25 bcd	14.72 ± 0.30 bcd	1.35 ± 0.00 jk	1.36 ± 0.00 hi	49.06 ± 0.01 g	48.68 ± 0.01 h
		Compost-5 + PGPR	12.25 ± 0.30 fghijk	12.28 ± 0.57 fghij	1.31 ± 0.00 n	1.28 ± 0.00 no	50.55 ± 0.02 c	51.68 ± 0.02 b
		Compost-10 + PGPR	10.80 ± 0.57 klmno	10.84 ± 0.52 klmno	1.29 ± 0.00 p	1.29 ± 0.00 n	51.32 ± 0.02 a	51.32 ± 0.02 c
	EC_e_ 6.3	Control	16.55 ± 0.52 a	16.69 ± 0.31 a	1.41 ± 0.01 b	1.45 ± 0.01 a	46.66 ± 0.25 o	45.15 ± 0.26 o
		Compost-5	14.13 ± 0.31 cde	14.26 ± 0.28 cde	1.37 ± 0.00 ef	1.41 ± 0.00 d	48.28 ± 0.03 kl	46.77 ± 0.03 l
		Compost-10	11.68 ± 0.29 ghijkl	11.91 ± 0.36 ghijk	1.37 ± 0.00 def	1.37 ± 0.00 f	48.25 ± 0.00 kl	48.25 ± 0.00 j
		PGPR	14.30 ± 0.62 cde	14.66 ± 0.74 bcd	1.39 ± 0.00 c	1.44 ± 0.00 b	47.54 ± 0.00 n	45.65 ± 0.00 n
		Compost-5 + PGPR	10.98 ± 0.72 klmn	11.35 ± 0.22 jklmn	1.36 ± 0.00 ghi	1.36 ± 0.00 ghi	48.66 ± 0.02 ij	48.66 ± 0.02 hi
		Compost-10 + PGPR	10.91 ± 0.21 klmno	11.32 ± 0.22 jklmn	1.34 ± 0.00 lm	1.33 ± 0.00 k	49.45 ± 0.02 ef	49.82 ± 0.01 f

Means followed by different letters are significant according to Tukey’s test at level of *p* ≤ 0.05. Note, double letter is different from single letter.

**Table 5:** Effects of compost and PGPR treatments on soil biological properties and nutrient availability in maize under salinity stress (2023–2024).

			Available N		Soil Organic Matter		Total Bacteria		Total Fungi	
			2023	2024	2023	2024	2023	2024	2023	2024
Variety		131	25.79 ± 0.03 a	27.23 ± 0.03 a	1.08 ± 0.00 a	1.09 ± 0.00 a	4.76 ± 0.23 a	5.25 ± 0.27 a	1.41 ± 0.05 c	1.49 ± 0.05 c
		132	25.79 ± 0.03 a	27.24 ± 0.03 a	1.08 ± 0.00 a	1.09 ± 0.00 a	4.72 ± 0.32 a	5.22 ± 0.35 a	1.47 ± 0.06 b	1.60 ± 0.07 b
		178	25.79 ± 0.02 a	27.24 ± 0.03 a	1.07 ± 0.00 a	1.09 ± 0.00 a	4.33 ± 0.23 b	4.76 ± 0.27 b	1.53 ± 0.06 a	1.67 ± 0.06 a
Salinity		EC 3.5	27.83 ± 0.03	29.26 ± 0.03	1.16 ± 0.00	1.17 ± 0.00	4.95 ± 0.32	5.46 ± 0.36	1.59 ± 0.08	1.71 ± 0.07
		EC 6.3	25.18 ± 0.02	26.65 ± 0.03	1.05 ± 0.00	1.06 ± 0.00	4.44 ± 0.28	4.90 ± 0.32	1.39 ± 0.05	1.50 ± 0.06
Treatments		Control	17.57 ± 0.08 f	18.60 ± 0.09 f	0.96 ± 0.00 c	0.93 ± 0.00 c	3.43 ± 0.37 c	3.82 ± 0.44 d	1.29 ± 0.07 e	1.38 ± 0.08 d
		Compost-5	23.71 ± 0.01 d	24.83 ± 0.01 d	1.00 ± 0.00 c	1.01 ± 0.00 b	4.29 ± 0.26 b	4.77 ± 0.31 c	1.39 ± 0.05 d	1.50 ± 0.05 c
		Compost-10	25.89 ± 0.04 c	28.11 ± 0.04 c	1.12 ± 0.00 b	1.14 ± 0.00 b	4.30 ± 0.21 b	4.76 ± 0.23 c	1.48 ± 0.08 c	1.60 ± 0.08 b
		PGPR	21.02 ± 0.00 e	21.59 ± 0.00 e	0.99 ± 0.00 b	1.01 ± 0.00 b	4.80 ± 0.24 b	5.30 ± 0.29 b	1.52 ± 0.08 b	1.61 ± 0.06 b
		Compost-5 + PGPR	30.63 ± 0.01 b	31.73 ± 0.01 b	1.19 ± 0.00 a	1.22 ± 0.00 a	5.39 ± 0.25 a	5.91 ± 0.26 a	1.57 ± 0.04 a	1.71 ± 0.04 a
		Compost-10 + PGPR	35.91 ± 0.02 a	38.56 ± 0.02 a	1.21 ± 0.00 a	1.24 ± 0.00 a	5.40 ± 0.22 a	5.92 ± 0.27 a	1.57 ± 0.04 a	1.72 ± 0.05 a
Interaction										
Maize variety	Salinity (dS/m)	Treatments								
Single Cross 131	EC_e_ 3.5	Control	19.26 ± 0.09 y	20.72 ± 0.10 v	1.08 ± 0.01 n	1.05 ± 0.00 p	4.10 ± 0.12 ghijklm	4.51 ± 0.15 ghijkl	1.33 ± 0.11 ijkl	1.26 ± 0.04 mnopq
		Compost-5	25.22 ± 0.01 o	26.17 ± 0.01 p	1.11 ± 0.00 m	1.12 ± 0.00 m	4.83 ± 0.13 defg	5.40 ± 0.07 defg	1.46 ± 0.04 bcdefghij	1.60 ± 0.04 fghijklmn
		Compost-10	28.30 ± 0.05 j	30.22 ± 0.05 k	1.21 ± 0.00 lf	1.24 ± 0.00 e	4.80 ± 0.17 defg	5.30 ± 0.16 defg	1.60 ± 0.11 abcde	1.70 ± 0.10 cdefghij
		PGPR	22.66 ± 0.01 s	23.33 ± 0.01 s	1.08 ± 0.00 n	1.11 ± 0.00 n	5.61 ± 0.34 bcd	6.15 ± 0.38 bcd	1.63 ± 0.11 abcd	1.72 ± 0.06 cdefghij
		Compost-5 + PGPR	33.37 ± 0.01 f	34.67 ± 0.01 f	1.24 ± 0.00 c	1.26 ± 0.00 c	6.81 ± 0.08 a	7.28 ± 0.28 a	1.66 ± 0.04 ab	1.76 ± 0.03 bcdefg
		Compost-10 + PGPR	38.62 ± 0.02 b	40.54 ± 0.02 a	1.26 ± 0.00 a	1.28 ± 0.00 a	6.41 ± 0.36 ab	7.06 ± 0.36 ab	1.58 ± 0.05 abcdefg	1.70 ± 0.02 cdefghijk
	EC_e_ 6.3	Control	15.55 ± 0.07 bb	17.01 ± 0.08 z	0.84 ± 0.00 x	0.82 ± 0.00 w	2.88 ± 0.48 n	3.19 ± 0.55 m	1.12 ± 0.01 l	1.19 ± 0.06 r
		Compost-5	21.90 ± 0.01 u	22.46 ± 0.01 u	0.88 ± 0.00 u	0.86 ± 0.00 u	3.65 ± 0.31 ijklmn	4.09 ± 0.38 hijklm	1.22 ± 0.03 kl	1.31 ± 0.08 qr
		Compost-10	23.69 ± 0.04 q	25.63 ± 0.05 r	1.02 ± 0.00 r	1.05 ± 0.00 p	3.75 ± 0.31 hijklmn	4.17 ± 0.37 hijklm	1.23 ± 0.03 kl	1.32 ± 0.06 pqr
		PGPR	19.07 ± 0.00 z	19.69 ± 0.00 y	0.88 ± 0.00 u	0.91 ± 0.00 s	4.52 ± 0.22 efghi	5.05 ± 0.35 defghi	1.32 ± 0.07 ijkl	1.36 ± 0.06 opqr
		Compost-5 + PGPR	29.07 ± 0.01 i	30.08 ± 0.01 l	1.17 ± 0.00 h	1.19 ± 0.00 i	4.84 ± 0.17 defg	5.37 ± 0.13 defg	1.36 ± 0.04 ghijk	1.49 ± 0.04 jklmnopq
		Compost-10 + PGPR	32.72 ± 0.02 g	36.26 ± 0.02 e	1.19 ± 0.00 g	1.20 ± 0.00 h	4.93 ± 0.10 defg	5.45 ± 0.09 defg	1.37 ± 0.03 fghijk	1.46 ± 0.04 lmnopq
Single Cross 132	EC_e_ 3.5	Control	19.33 ± 0.09 y	20.19 ± 0.09 x	1.04 ± 0.00 p	1.01 ± 0.00 q	3.50 ± 0.46 jklmn	3.96 ± 0.66 ijklm	1.26 ± 0.06 jkl	1.41 ± 0.12 mnopqr
		Compost-5	26.56 ± 0.01 m	27.42 ± 0.01 o	1.14 ± 0.00 k	1.16 ± 0.00 k	4.57 ± 0.36 efghi	5.10 ± 0.40 defgh	1.40 ± 0.08 efghijk	1.49 ± 0.05 ijklmnopq
		Compost-10	27.31 ± 0.05 l	31.02 ± 0.05 i	1.22 ± 0.00 e	1.23 ± 0.00 f	4.36 ± 0.27 fghijkl	4.85 ± 0.35 fghij	1.57 ± 0.10 abcdefg	1.67 ± 0.14 cdefghijkl
		PGPR	22.07 ± 0.00 t	23.32 ± 0.00 s	1.08 ± 0.00 n	1.09 ± 0.00 o	5.18 ± 0.51 cdef	5.77 ± 0.59 cdef	1.59 ± 0.09 abcdef	1.72 ± 0.09 cdefghi
		Compost-5 + PGPR	32.27 ± 0.01 h	33.83 ± 0.01 g	1.24 ± 0.00 c	1.25 ± 0.00 d	6.13 ± 0.67 abc	6.71 ± 0.56 ab	1.72 ± 0.05 a	1.85 ± 0.06 abcd
		Compost-10 + PGPR	40.08 ± 0.02 a	40.63 ± 0.02 a	1.26 ± 0.00 a	1.27 ± 0.00 b	6.21 ± 0.23 ab	6.73 ± 0.33 ab	1.66 ± 0.07 ab	1.80 ± 0.09 abcdef
	EC_e_ 6.3	Control	15.79 ± 0.04 aa	16.26 ± 0.08 aa	0.86 ± 0.00 w	0.84 ± 0.00 v	3.47 ± 0.28 klmn	3.88 ± 0.44 jklm	1.26 ± 0.05 ijkl	1.38 ± 0.04 nopqr
		Compost-5	20.86 ± 0.01 w	22.75 ± 0.01 t	0.87 ± 0.00 v	0.89 ± 0.00 t	4.43 ± 0.19 efghijk	4.91 ± 0.35 efghij	1.28 ± 0.06 ijkl	1.40 ± 0.04 mnopqr
		Compost-10	24.06 ± 0.04 p	25.81 ± 0.04 q	1.04 ± 0.00 p	1.05 ± 0.00 p	4.29 ± 0.19 fghijkl	4.70 ± 0.17 fghijkl	1.41 ± 0.08 efghijk	1.54 ± 0.09 ghijklmnop
		PGPR	19.81 ± 0.00 x	20.09 ± 0.00 x	0.91 ± 0.00 s	0.92 ± 0.00 r	4.89 ± 0.12 defg	5.37 ± 0.05 defg	1.42 ± 0.03 defghijk	1.55 ± 0.02 ghijklmnop
		Compost-5 + PGPR	27.58 ± 0.01 k	28.24 ± 0.01 m	1.16 ± 0.00 i	1.17 ± 0.00 j	4.87 ± 0.06 defg	5.39 ± 0.06 defg	1.48 ± 0.06 bcdefghi	1.61 ± 0.08 efghijklmn
		Compost-10 + PGPR	33.69 ± 0.02 e	37.27 ± 0.02 c	1.15 ± 0.00 j	1.21 ± 0.00 g	4.78 ± 0.28 defg	5.32 ± 0.29 defg	1.60 ± 0.01 abcde	1.73 ± 0.02 cdefgh
Single Cross 178	EC_e_ 3.5	Control	19.92 ± 0.09 x	20.38 ± 0.10 w	1.04 ± 0.00 p	1.01 ± 0.00 q	3.26 ± 0.57 mn	3.63 ± 0.56 lm	1.56 ± 0.11 abcdefgh	1.70 ± 0.13 cdefghij
		Compost-5	26.22 ± 0.01 n	27.70 ± 0.01 n	1.11 ± 0.00 m	1.13 ± 0.00 l	4.19 ± 0.50 fghijklm	4.69 ± 0.55 fghijkl	1.65 ± 0.09 abc	1.76 ± 0.06 bcdefg
		Compost-10	28.25 ± 0.05 j	30.20 ± 0.05 kl	1.21 ± 0.00 f	1.23 ± 0.00 f	4.50 ± 0.23 efghij	4.94 ± 0.26 efghij	1.73 ± 0.08 a	1.88 ± 0.02 abc
		PGPR	23.46 ± 0.00 r	23.41 ± 0.00 s	1.06 ± 0.00 o	1.09 ± 0.00 o	4.38 ± 0.26 fghijkl	4.83 ± 0.29 fghijk	1.76 ± 0.13 a	1.84 ± 0.13 abcde
		Compost-5 + PGPR	32.38 ± 0.01 h	32.95 ± 0.01 h	1.23 ± 0.00 d	1.25 ± 0.00 d	4.77 ± 0.13 defg	5.29 ± 0.23 defg	1.77 ± 0.02 a	1.98 ± 0.01 ab
		Compost-10 + PGPR	35.62 ± 0.02 c	40.02 ± 0.02 b	1.25 ± 0.00 b	1.27 ± 0.00 b	5.43 ± 0.22 bcde	6.02 ± 0.37 bcde	1.74 ± 0.06 a	2.00 ± 0.09 a
	EC_e_ 6.3	Control	15.55 ± 0.07 bb	17.01 ± 0.08 z	0.86 ± 0.00 w	0.84 ± 0.00 v	3.39 ± 0.16 lmn	3.74 ± 0.27 klm	1.22 ± 0.11 kl	1.35 ± 0.06 pqr
		Compost-5	21.50 ± 0.01 v	22.46 ± 0.01 u	0.87 ± 0.00 v	0.89 ± 0.00 t	4.05 ± 0.06 ghijklm	4.44 ± 0.11 ghijkl	1.31 ± 0.03 ijkl	1.41 ± 0.07 mnopqr
		Compost-10	23.72 ± 0.00 q	25.77 ± 0.00 q	1.03 ± 0.00 q	1.05 ± 0.00 p	4.13 ± 0.08 ghijklm	4.60 ± 0.06 ghijkl	1.35 ± 0.07 hijk	1.48 ± 0.06 klmnopq
		PGPR	19.05 ± 0.00 z	19.70 ± 0.00 y	0.90 ± 0.00 t	0.92 ± 0.00 r	4.22 ± 0.04 fghijklm	4.61 ± 0.06 ghijkl	1.40 ± 0.04 efghijk	1.50 ± 0.01 hijklmnopq
		Compost-5 + PGPR	29.07 ± 0.01 i	30.60 ± 0.01 j	1.12 ± 0.00 l	1.17 ± 0.00 j	4.93 ± 0.38 defg	5.42 ± 0.27 defg	1.43 ± 0.03 cdefghijk	1.58 ± 0.04 fghijklmno
		Compost-10 + PGPR	34.70 ± 0.01 d	36.66 ± 0.01 d	1.15 ± 0.00 j	1.21 ± 0.00 g	4.66 ± 0.13 defgh	4.95 ± 0.18 efghij	1.46 ± 0.02 bcdefghij	1.62 ± 0.04 defghijklm

Means followed by different letters are significant according to Tukey’s test at level of *p* ≤ 0.05. Note, double letter is different from single letter.

**Table 6:** Properties of the experimental soil during the 2023 and 2024 seasons.

Parameter	Soil 1 (Control)		Soil 2 (Saline)	
	2023	2024	2023	2024
pH (1:2.5 soil: distilled water suspension)	7.47 ± 0.01	7.67 ± 0.01	8.15 ± 0.02	8.20 ± 0.01
EC ^¥^ (Soil paste extract; dS/m)	3.70 ± 0.03	3.28 ± 0.02	6.23 ± 0.03	6.41 ± 0.02
Soluble ions (meq/L)				
Na^+^	19.86 ± 0.95	20.86 ± 0.95	26.78 ± 0.84	26.78 ± 0.84
K^+^	0.27 ± 0.01	0.33 ± 0.01	0.53 ± 0.01	0.54 ± 0.01
Ca^2+^	7.02 ± 0.51	7.09 ± 0.51	9.94 ± 0.42	9.99 ± 0.42
Mg^2+^	6.65 ± 0.33	6.75 ± 0.33	7.62 ± 0.25	7.60 ± 0.25
CO_3_^2−^	nd ^†^	nd	nd	nd
HCO_3_^−^	10.24 ± 0.72	10.24 ± 0.72	10.75 ± 0.61	10.72 ± 0.61
Cl^−^	14.26 ± 0.88	14.26 ± 0.88	23.05 ± 0.83	22.05 ± 0.83
SO_4_^2−^	9.30 ± 0.31	9.30 ± 0.31	9.69 ± 0.22	9.79 ± 0.22
SAR (Sodium adsorption ratio)	9.59 ± 0.12	9.09 ± 0.11	12.58 ± 0.14	12.58 ± 0.15
Exchangeable sodium percentage (%)	11.41 ± 0.11	10.83 ± 0.09	14.75 ± 0.14	14.74 ± 0.13
Available macronutrients (mg/kg)				
N	39.67 ± 1.21	40.23 ±1.31	21.36 ± 1.11	28.29 ± 1.24
P	4.48 ± 0.21	4.51 ± 0.25	2.86 ± 0.15	2.98 ± 0.19
K	336 ± 21	335 ± 25	215 ± 21	204 ± 25
Bulk density (kg/m^3^)	1.37 ± 0.01	1.38 ± 0.02	1.42 ± 0.01	1.44 ± 0.02
Total porosity (%)	48.30 ± 3.41	47.92 ± 2.32	46.42 ± 3.19	45.66 ± 3.21
Organic matter (%)	1.06 ± 0.01	1.04 ± 0.01	0.85 ± 0.02	0.84 ± 0.01
CaCO_3_ (%)	2.13 ± 0.02	2.12 ± 0.01	2.95 ± 0.02	2.94 ± 0.02
Field capacity (%)	42.43 ± 0.32	43.45 ±0.23	40.73 ± 0.31	40.42 ± 0.29
Wilting point (%)	22.21 ± 0.22	22.34 ± 0.20	21.53 ± 0.22	21.47 ± 0.19
Cation exchange capacity (cmolc/kg)	1.86 ± 0.01	1.96 ± 0.01	1.56 ± 0.01	1.55 ± 0.01
Sand (%)	15.56 ±0.11	15.65 ±0.10	15.49 ± 0.11	15.07 ± 0.12
Silt (%)	32.11 ±0.18	32.48 ±0.16	32.19 ± 0.18	32.56 ± 0.19
Clay (%)	52.33 ±0.19	51.87 ±0.17	52.32 ± 0.21	52.37 ± 0.22
Texture class	Clay	Clay	Clay	Clay
Soil classification	alluvial	alluvial	alluvial	alluvial

^¥^ Electrical conductivity; ^†^ not detected.

**Table 7:** Properties of the compost applied in the experiment in 2023 and 2024 seasons.

Parameter	2023	2024
pH (1:10 compost: distilled water suspension)	6.77 ± 0.01	6.79 ± 0.01
EC ^¥^ (1:10 compost: distilled water extract; dS/m)	4.51 ± 0.05	4.57 ± 0.04
Organic matter (%)	38.84 ± 2.22	38.82 ± 1.98
N (%)	1.54 ± 0.03	1.52 ± 0.04
C (%)	33.54 ± 0.92	32.95 ± 0.88
C:N	23.11 ± 1.01	23.09 ± 0.99
P (%)	0.88 ± 0.02	0.87 ± 0.03
K (%)	1.41 ± 0.13	1.46 ± 0.11
Manganese (mg/kg)	361 ± 25	369 ± 26
Iron (mg/kg)	349 ± 32	341 ± 27
Zinc (mg/kg)	72 ± 11	70 ± 10

^¥^ Electrical conductivity.

## Data Availability

Data are contained within the article.

## Abbreviations

PGPR	Plant Growth-Promoting Rhizobacteria
SAR	Sodium Adsorption Ratio
SOM	Soil Organic Matter
ESP	Exchangeable Sodium Percentage
Ava-N	Available nitrogen
